# Targeting interleukin-6 as a strategy to overcome stroma-induced resistance to chemotherapy in gastric cancer

**DOI:** 10.1186/s12943-019-0972-8

**Published:** 2019-03-30

**Authors:** In-Hye Ham, Hye Jeong Oh, Hyejin Jin, Cheong A Bae, Sang-Min Jeon, Kyeong Sook Choi, Sang-Yong Son, Sang-Uk Han, Rolf A. Brekken, Dakeun Lee, Hoon Hur

**Affiliations:** 1 Department of Surgery, Ajou University School of Medicine, 164 Worldcup-ro, Yeongtong-gu, Suwon-si, 16499 Gyeonggi-do Republic of Korea; 2 Department of Biomedical Sciences, Ajou University Graduate School of Medicine, 164 Worldcup-ro, Yeongtong-gu, Suwon-si, 16499 Gyeonggi-do Republic of Korea; 3 Department of Pharmacy, Ajou University College of Pharmacy, 206 Worldcup-ro, Yeongtong-gu, Suwon-si, 16499 Gyeonggi-do Republic of Korea; 4 Department of Biochemistry and Molecular Biology, Ajou University School of Medicine, 164 Worldcup-ro, Yeongtong-gu, Suwon-si, 16499 Gyeonggi-do Republic of Korea; 5 Division of Surgical Oncology, Department of Surgery, Hamon Center for Therapeutic Oncology Research, University of Texas Southwestern Medical Center, Dallas, TX 75390 USA; 6 Department of Pathology, Ajou University School of Medicine, 164 Worldcup-ro, Yeongtong-gu, Suwon-si, Gyunggi-do 16499 Republic of Korea

**Keywords:** Gastric cancer, Cancer-associated fibroblasts, Tumor microenvironment, Chemo-resistance, Interleukin-6, Tocilizumab, Jak1-STAT3

## Abstract

**Background:**

Although the tumor stroma in solid tumors like gastric cancer (GC) plays a crucial role in chemo-resistance, specific targets to inhibit the interaction between the stromal and cancer cells have not yet been utilized in clinical practice. The present study aims to determine whether cancer-associated fibroblasts (CAFs), a major component of the tumor stroma, confer chemotherapeutic resistance to GC cells, and to discover potential targets to improve chemo-response in GC.

**Methods:**

To identify CAF-specific proteins and signal transduction pathways affecting chemo-resistance in GC cells, secretome and transcriptome analyses were performed. We evaluated the inhibiting effect of CAF-specific protein in in vivo and in vitro models and investigated the expression of CAF-specific protein in human GC tissues.

**Results:**

Secretome and transcriptome data revealed that interleukin-6 (IL-6) is a CAF-specific secretory protein that protects GC cells via paracrine signaling. Furthermore, CAF-induced activation of the Janus kinase 1-signal transducer and activator of transcription 3 signal transduction pathway confers chemo-resistance in GC cells. CAF-mediated inhibition of chemotherapy-induced apoptosis was abrogated by the anti-IL-6 receptor monoclonal antibody tocilizumab in various experimental models. Clinical data revealed that IL-6 was prominently expressed in the stromal portion of GC tissues, and IL-6 upregulation in GC tissues was correlated with poor responsiveness to chemotherapy.

**Conclusions:**

Our data provide plausible evidence for crosstalk between GC cells and CAFs, wherein IL-6 is a key contributor to chemoresistance. These findings suggest the potential therapeutic application of IL-6 inhibitors to enhance the responsiveness to chemotherapy in GC.

**Electronic supplementary material:**

The online version of this article (10.1186/s12943-019-0972-8) contains supplementary material, which is available to authorized users.

## Background

Gastric cancer (GC) is the fifth most common malignancy and the third leading cause of cancer-related mortalities worldwide [1]. Systemic chemotherapy with multiple drug regimens may be the only treatment option for patients with recurrent and metastatic GC. In addition, the benefits of palliative chemotherapy and supportive care have been reported to demonstrate limited response rates of 25 to 50% and median survival times of 6 to 12 months [2–4].

Numerous previous studies have reported that chemotherapeutic resistance in solid tumors such as those in GC results from individual variations among patients and genetic heterogeneity among tumor cells. In addition, treatment-induced upregulation of genes including those associated with multi-drug resistance (MDR) or multidrug resistance protein (MRP) enhances chemotherapeutic resistance in cancer cells [5, 6]. However, these studies have focused on the intrinsic pathways in cancer cells. Recently, the function of the tumor stroma in chemotherapeutic resistance has attracted attention. During carcinogenesis, cancer-associated fibroblasts (CAFs), which differ from normal fibroblasts phenotypically and functionally, are activated as a major component of the tumor stroma [7]. The interaction of CAFs with tumor cells may contribute to aggressive phenotypes of cancer cells, including the development of metastatic potential and chemotherapeutic resistance [8]. Molecular analyses have revealed a close correlation between the accumulation of activated fibroblasts within tumors and poor response to chemotherapy in GC [9, 10], but the mechanism by which CAFs contribute to chemotherapeutic resistance is not clear. Moreover, the efficacy of molecular inhibitors in suppressing CAF-mediated chemotherapeutic resistance in cancer has yet not been assessed clinically.

Interleukin-6 (IL-6), a multifaceted cytokine that mediates responses to injury or infection, is also involved in immune diseases and cancers [11–13]. In cancers, IL-6 is produced by cancer cells and inflammatory and stromal cells. Since extracellular IL-6 binds to the cell surface receptor glycoprotein 130 (gp130) and consequently activates several cell-survival-related pathways, several studies have investigated the function of IL-6 in promoting chemotherapeutic resistance in various cancers [11, 14, 15]; however, only a few studies have focused on the role of IL-6 produced by stromal cells in the tumor microenvironment [16, 17]. While previous studies have implicated the stroma in the aggressiveness of GC [18, 19], the function of IL-6 produced from CAFs in the development of chemotherapeutic resistance has not yet been evaluated.

In the present study, through in vitro and in vivo studies and bioinformatic analysis of clinical data, we provide evidence that IL-6 produced by CAFs is a critical contributor to chemoresistance in GC.

## Methods

### Cell lines and cell culture

We purchased the GC cell lines MKN-1 (KCLB No. 80101) and MKN-45 (KCLB No. 80103) from the Korean Cell Line Bank (Seoul, Republic of Korea). Further details are provided in Additional file 1.

### Isolation and culturing of fibroblasts

Human GC specimens were obtained from patients undergoing tumor resection surgery at the Ajou University Hospital (Suwon, Republic of Korea). Fibroblasts were isolated from their GC tissues (CAFs) and paired normal tissues (NAFs), as described in Additional file 1.

### Co-culture with CAFs or NAFs

MKN-1 and MKN-45 cells were seeded into the bottom of 6-well transwell chamber plates (Corning, Union City, CA, USA) at a density of 1 × 10^5^ cells/well, and then, NAFs or CAFs were seeded onto the upper insert membrane (0.4-μm pore size) of the chamber. Further details are provided in Additional file 1.

### Western blotting

The cells were washed with phosphate-buffered saline and lysed in lysis buffer. The lysates were incubated on ice for 20 min and centrifuged at 13,000 rpm for 20 min at 4 °C. Samples with equalized protein concentrations were subjected to SDS-PAGE and electroblotted onto polyvinylidene difluoride membranes (Millipore, Billerica, MA, USA). Additional details, including the antibodies used, are provided in Additional file 1.

### Secretome analysis

We performed secretome analysis to identify the upregulated secretory factors in the culture supernatants of MKN-45 cells co-cultured with CAFs, relative to that in the culture supernatants of MKN-45 cells not co-cultured with CAFs. We used a Proteome Profiler Human Cytokine Array Kit (R&D Systems Inc., Minneapolis, MN, USA). A more detailed description is provided in Additional file 1.

### Reverse transcriptase PCR (RT-PCR)

Total RNA extracted from monocytes, GC cells, and fibroblasts was converted to cDNA using 1 μg of RNA from each cell type as the template, in a final volume of 20 μl. A detailed description is provided in Additional file 1.

### Quantitative RT-PCR (qRT-PCR)

Total RNA was isolated using a Total RNA Isolation Kit (Qiagen, Hilden, Germany) in accordance with the manufacturer’s instructions. We generated cDNA using 1 μg of total RNA as a template, with a cDNA Synthesis Master Mix Kit (GenDEPOT, Barker, TX, USA). A detailed description is provided in Additional file 1.

### Enzyme-linked immunosorbent assay (ELISA)

IL-6 concentrations in the cultured media of GC cells and fibroblasts were measured using a Human IL-6 Quantikine ELISA Kit (R&D Systems, Minneapolis, MN, USA) according to the manufacturer’s instructions.

### Cell viability assay

Cells in each group were seeded into 96-well culture plates (10,000–15,000 cells/well) and incubated for 72 h at 37 °C with 5% CO_2_ and 95% O_2_. Thereafter, Ez-Cytox reagent (Deaillab, Seoul, Republic of Korea) was added to each well, followed by incubation for 1–2 h at 37 °C. A more detailed description is provided in Additional file 1.

### Immunohistochemical staining

Formalin-fixed paraffin-embedded human or xenograft tumors were sectioned, affixed onto microscope slides, deparaffinized with xylene, hydrated using a diluted alcohol series, and immersed in 0.3% H_2_O_2_ in methanol to quench the endogenous peroxidase activity. More details, including the antibodies used, are described in previous our paper [20] and Additional file 1.

### Immunofluorescence staining

Paraffin-embedded sections from MKN-1 and MKN-45 xenograft tumors were deparaffinized with xylene, hydrated using a diluted alcohol series, and immersed in 0.3% H_2_O_2_ in methanol to quench the endogenous peroxidase activity. More details, including the antibodies used, are provided in previous our paper [20] and Additional file 1.

### Generation of inducible short hairpin (sh) RNA for IL-6 (shIL-6)

To generate shRNA-expressing plasmids, double-stranded oligos encoding the desired shRNA were cloned into the single-vector inducible shRNA construct pLKO-Tet-On. A more detailed description is provided in Additional file 1.

### Animal study

The animal care and handling procedures were performed in accordance with the Ajou University School of Medicine’s Institutional Animal Care and Use Committee guidelines, and all animal experiments were approved by the Animal Research Committee of the institution (IACUC protocol 2015–0069). A more detailed description is provided in Additional file 1.

### The Cancer genome atlas (TCGA) data

We analyzed the TCGA stomach adenocarcinoma dataset using the cBioportal tool (http://www.cbioportal.org). A more detailed description is provided in Additional file 1.

### Gene expression analysis in the biopsied GC tissues

To assess the differential gene expression in biopsy samples in response to chemotherapy, 10 GC patients treated preoperatively with 5-fluorouracil (5-FU)-based chemotherapies, followed by surgical resection were included in this study. This study was approved by the institutional review board/ethics committee of the Ajou University Hospital (AJIRB-BMR-KSP-15-432). A more detailed description is provided in Additional file 1.

### Statistical analysis

A detailed description of the statistical analyses used is provided in Additional file 1.

## Results

### Fibroblasts reduce the responsiveness of GC cells to 5-FU

**Fig. 1:**
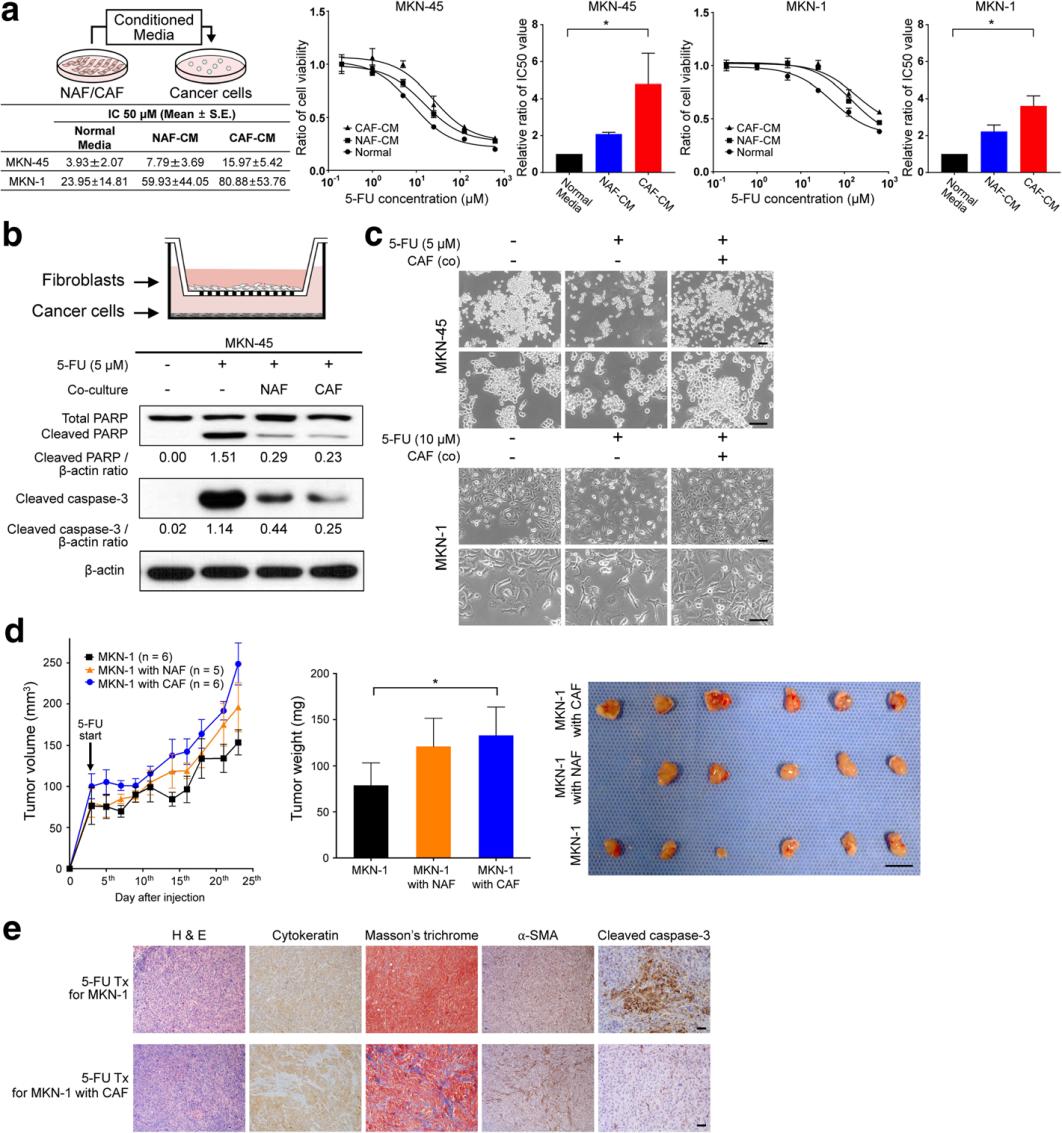
Cancer-associated fibroblast (CAF)-induced resistance to 5-fluorouracil (5-FU) in gastric cancer cells. **a** MKN-45 and MKN-1 gastric cancer cells treated with 5-FU were treated with fibroblast culture-conditioned media, and the half maximal inhibitory concentration (IC_50_) was measured. The results are presented as the mean (± SEM). *P < 0.05, based on Kruskal-Wallis test followed by a Dunn’s multiple comparison. **b** Schematic figure detailing the transwell co-culture system with fibroblasts isolated from paired normal gastric tissues (normal-associated fibroblasts or NAFs) and gastric cancer tissues (CAFs). Western blot analysis results show changes in the expression of apoptotic markers such as cleaved PARP and caspase-3 72 h after 5-FU treatment with and without co-culture with NAFs and CAFs. **c** Representative micrographs demonstrating morphological changes in MKN-45 and MKN-1 cells after 5-FU treatment for 72 h with and without co-culture with fibroblasts. Scale bar = 100 μm. **d** A line graph comparing tumor growth among the in vivo xenograft tumors derived from MKN-1 cells alone (*n* = 6), MKN-1 cells combined with NAFs (*n* = 5), or MNK-1 cells combined with CAFs (*n* = 5) after 5-FU treatments. The bar graph compares the harvested tumor weight among the three groups. Graphs show the mean (± SEM) tumor weights of the mice. ^*^P < 0.05, based on one-way ANOVA analysis, followed by a post hoc test with Tukey’s method. The photographs show the harvested tumors. Scale bar = 1 cm. **e**. Representative micrographs showing H&E staining, Masson’s trichrome staining for stromal collagen fibers, and immunohistochemical staining for α-smooth muscle actin (a-SMA), cytokeratin, and cleaved caspase-3 in harvested xenograft tumors derived from only MKN-1 cells and those derived from MKN-1 cells mixed with CAFs after treatment with 5-FU. Scale bar = 100 μm

To investigate the paracrine effect of CAFs on the responsiveness of GC cell lines to chemotherapy, conditioned medium (CM) from NAF and CAF cultures was added to MKN-45 and MKN-1 cells treated with 5-FU (Fig. 1a). Cell viability assays showed that MKN-45 cells incubated with CAF-CM showed increased resistance (greater IC_50_) to 5-FU than MKN-45 cells treated with NAF-CM or control media (*P = 0.004*). Similar results were observed for MKN-1 cells (*P = 0.007*) (Fig. 1a). CM from CAFs also enhanced the viability of GC cell lines treated with cisplatin (Additional file 2: Figure S1a).

When we examined the effect of NAFs and CAFs on the sensitivity of GC cell lines to 5-FU using a transwell co-culture system, co-culture with these fibroblasts was found to reduce the expression of apoptotic markers, including cleaved caspase-3 and PARP, with the CAFs showing a greater effect than the NAFs (Fig. 1b). In addition, while GC cell lines treated with 5-FU revealed apoptotic morphologies in the GC cell lines, co-culture with fibroblasts potently inhibited 5-FU-induced apoptotic phenotypes (Fig. 1c).

To determine the effect of fibroblasts on the resistance of GC to 5-FU in vivo, mice with xenografted tumors harboring only MKN-1 cells (1 × 10^6^ cells) or mice xenografted with MKN cells (1 × 10^6^ cells) together with NAFs or CAFs (1 × 10^5^ cells each) were treated with 5-FU. We found that the addition of NAFs or CAFs suppressed the anti-cancer effect of 5-FU in mice xenografted with MKN-1 cells. The mean weight of the extracted tumors after treatment was significantly higher for the xenografts mixed with CAFs than that of the tumors composed of only cancer cells (*P* = 0.020). Tumors mixed with NAFs also showed a larger size compared to the tumors with only cancer cells, but the differences were not significant (*P* = 0.085) (Fig. 1d). Immunohistochemical staining of the harvested tumors revealed that the CAF-mixed tumors contained more α-smooth muscle actin (SMA)-positive cells and stromal collagen fibers, and fewer cleaved caspase-3-positive cells within tumors, compared to those containing only cancer cells (Fig. 1e). Similar results were obtained from an in vivo model using MKN-45 cells (Additional file 2: Figure S1b). These results suggest that CAF confers 5-FU resistance to GC cell lines through the inhibition of apoptosis.

### CAF-secreted IL-6 activates the Janus kinase 1-signal transducer and activator of transcription 3 signal transduction pathway in GC cell lines

**Fig. 2:**
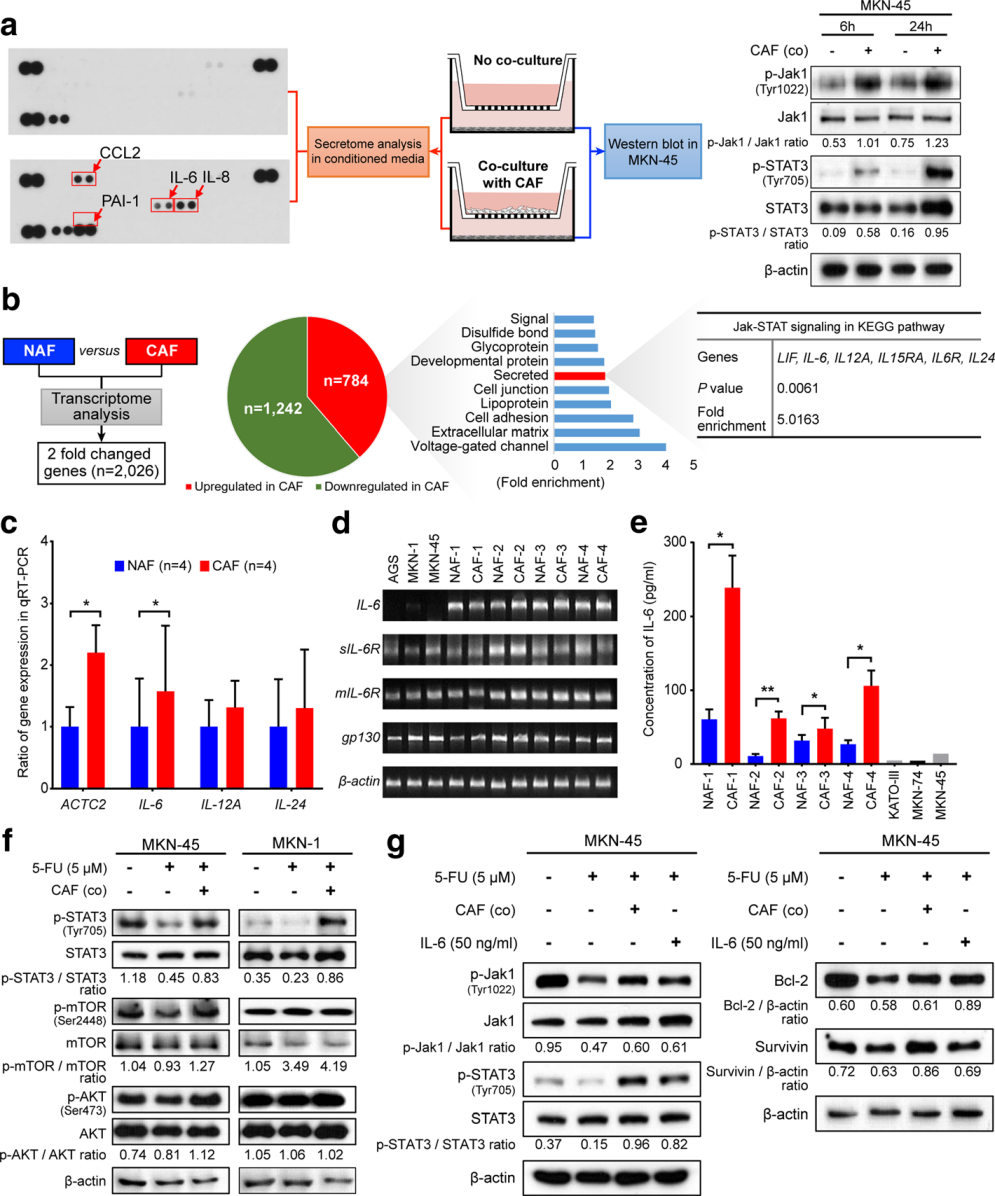
Identification of the IL-6/Jak1/STAT3 axis as a specific communicator between cancer-associated fibroblasts (CAFs) and gastric cancer cells. **a** Interleukin-6 (IL-6), interleukin-8 (IL-8), and C-C motif chemokine ligand 2 (CCL2) were secreted at higher levels in the media after co-culture with CAFs and MKN-45 cells than in the media used for culturing MKN-45 cells alone. Each of these factors were correlated with the Jak-STAT3 signal transduction pathway. The Western blot analysis shows expression changes of the indicated proteins with and without co-culture with CAFs for 6 or 24 h. **b** A flow chart depicting the transcriptome analysis of one paired set of normal-associated fibroblasts (NAFs) and CAFs. The pie graph presents the number of upregulated and downregulated genes in CAFs compared with those in NAFs. The graph and table show the functional annotation of results for 784 upregulated genes in CAFs from the Database for Annotation, Visualization, and Integrated Discovery (DAVID: https://david.ncifcrf.gov), which is based on the Kyoto Encyclopedia of Genes and Genomes pathways. **c** Results from quantitative PCR (qPCR) analysis showing the comparative mRNA expression of *ACTC2*, *IL6*, *IL12A*, and *IL24* between the NAFs and CAFs. The graphs show the mean (± SEM) ratio of mRNA expression in CAFs compared to those in NAFs. ^*^P < 0.05, based on paired t-tests. **d** Reverse transcription PCR (RT-PCR) results showing mRNA expression of IL-6 and its receptors in cells from the gastric cancer cell lines AGS, MKN-1, and MKN-45, and four paired NAFs and CAFs. **e** ELISA results showing IL-6 levels in the conditioned media from four paired NAFs and CAFs and from cells of the gastric cancer cell lines KATO-III, MKN-28, and MKN-45. ^*^P < 0.05 and ^**^P < 0.001, according to paired t-tests. **f, g** Western blot analysis showing the expression levels of the indicated proteins after 5-fluorouracil (5-FU) treatment (5 μM) with and without co-culture with CAFs and with and without recombinant IL-6 treatment in MKN-45 cells and MKN-1 cells

To identify CAF-specific secreted molecules that confer 5-FU resistance to cancer cells, we first investigated the release of 36 different cytokines, growth factors, and other proteins secreted into the CM of MKN-45 cells co-cultured with and without CAFs after 48 h of culture (Fig. 2a). We found that that several cytokines, including IL-6, IL-8, and chemokine (C-C motif) ligand 2 (CCL2), which are known to activate the Janus kinase 1-signal transducer and activator of transcription 3 (Jak1-STAT3) signaling pathway, were more abundantly present in the media co-cultured with CAFs and MKN-45 cells, compared to the culture media of the MKN-45 cells alone. We also found that p-Jak1 and p-STAT3 levels in MKN-45 cells increased progressively after co-culture with CAFs (Fig. 2b). Moreover, when we performed transcriptome analysis to compare the gene expression patterns between the paired NAF and CAF samples isolated from one GC patient, 784 genes were found to be upregulated in CAFs compared to NAFs, and 1242 genes were found to be downregulated, based on absolute fold changes of > 2 (Fig. 2b). To identify enriched function-related gene groups, the Database for Annotation, Visualization, and Integrated Discovery (https://david.ncifcrf.gov), which is based on the Kyoto Encyclopedia of Genes and Genomes (KEGG) pathways, was used. As a result, 114 genes encoding secretory proteins were found to be significantly enriched (*P* < 0.001; Fig. 2b). When we further performed gene functional analysis for these 114 genes that encoded secretory proteins, they were found to be significantly enriched in the Jak1-STAT3 signaling pathway and for several interleukin genes such as *IL6*, *IL12A*, and *IL24* that are involved in this pathway (Fig. 2b). We next compared the differential expression of these genes among the paired CAFs and NAFs isolated from four GC patients using qRT-PCR. In addition, in four paired NAFs and CAFs, we analyzed the RNA expression of α-SMA, a marker of activated fibroblasts. As expected, ACTA2 expression was significantly higher in CAFs than in NAFs (*P* = 0.013). Furthermore, *IL6* expression increased significantly in CAFs compared to NAFs (*P* = 0.018), while *IL12A* (*P* = 0.572) and *IL24* (*P* = 0.785) expression did not significantly increase (Fig. 2c). When we performed qPCR in different human GC cell lines and fibroblasts, interestingly, *sIL6R*, *mIL6*, and *gp130* mRNAs were expressed in cancer cells and paired fibroblasts, whereas *IL6* mRNA was expressed almost exclusively in fibroblasts (Fig. 2d). We further performed ELISA to measure the concentration of IL-6 in the culture media of the cancer cells KATO-III, MKN-28, and MKN-45, and fibroblasts. As expected, all CAFs displayed significantly higher levels of IL-6 secretion than their respective paired NAFs (NAF1 vs. CAF1, *P* = 0.018; NAF2 vs. CAF2, *P* = 0.006; NAF3 vs. CAF3, *P* = 0.038; NAF4 vs. CAF4, *P* = 0.021), while GC cells secreted very little IL-6 (Fig. 2e). To validate the results of the bioinformatics analysis in our experimental settings, we assessed whether CAFs actually activate Jak1 and STAT3 in GC cell lines. Western blot analysis revealed that co-culturing with CAFs increased the phosphorylation levels of Jak1 and STAT3, but not those of mTOR and Akt in cells from the GC cell lines MKN-45 and MKN-1 (Fig. 2f).

Finally, the expression of p-Jak1 and p-STAT3 in MKN-45 and MKN-1 cells treated with 5-FU was significantly higher when they were co-cultured with CAFs, compared to when they were not. Similarly, when GC cells were treated with recombinant IL-6 (50 ng/ml), the expression of p-Jak1, p-STAT3 and the expression of the anti-apoptosis markers Bcl-2 and survivin increased in MKN-45 and MKN-1 cells (Fig. 2g). To investigate whether CAFs also upregulate IL-6 expression in an in vivo xenograft model, we performed the immunohistochemistry of IL-6 using the tumor tissues from mice treated with 5-FU. Similar to the findings from the in vitro analyses, IL-6 expression was higher in CAF-mixed tumors than in tumors with only MKN-1 cells (Additional file 3: Figure S2a). These results suggest that in the microenvironment of GC tumors, IL-6 may originate primarily from CAFs and activate the Jak1-STAT3 pathway of GC cells via paracrine signaling.

To determine whether cancer cells affected IL-6 expression in the CAFs, we co-cultured CAFs with GC cells and evaluated the *IL6* mRNA expression using qRT-PCR. The expression of *IL6* mRNA was not significantly altered in CAFs co-cultured with GC cells (Additional file 3: Figure S2b). The ELISA and Western blot analyses revealed that neither co-culture with cancer cells nor 5-FU treatment increased the expression of IL-6 as well as NF-κB, a transcription factor for IL-6, in CAFs (Additional file 3: Figure S2c and d). These results suggest that IL-6 expression in the CAFs was not affected by co-culture with cancer cells or chemotherapeutic exposure.

### Inhibition of the IL-6/Jak1/STAT3 axis suppresses the drug resistance in GC cell lines

**Fig. 3:**
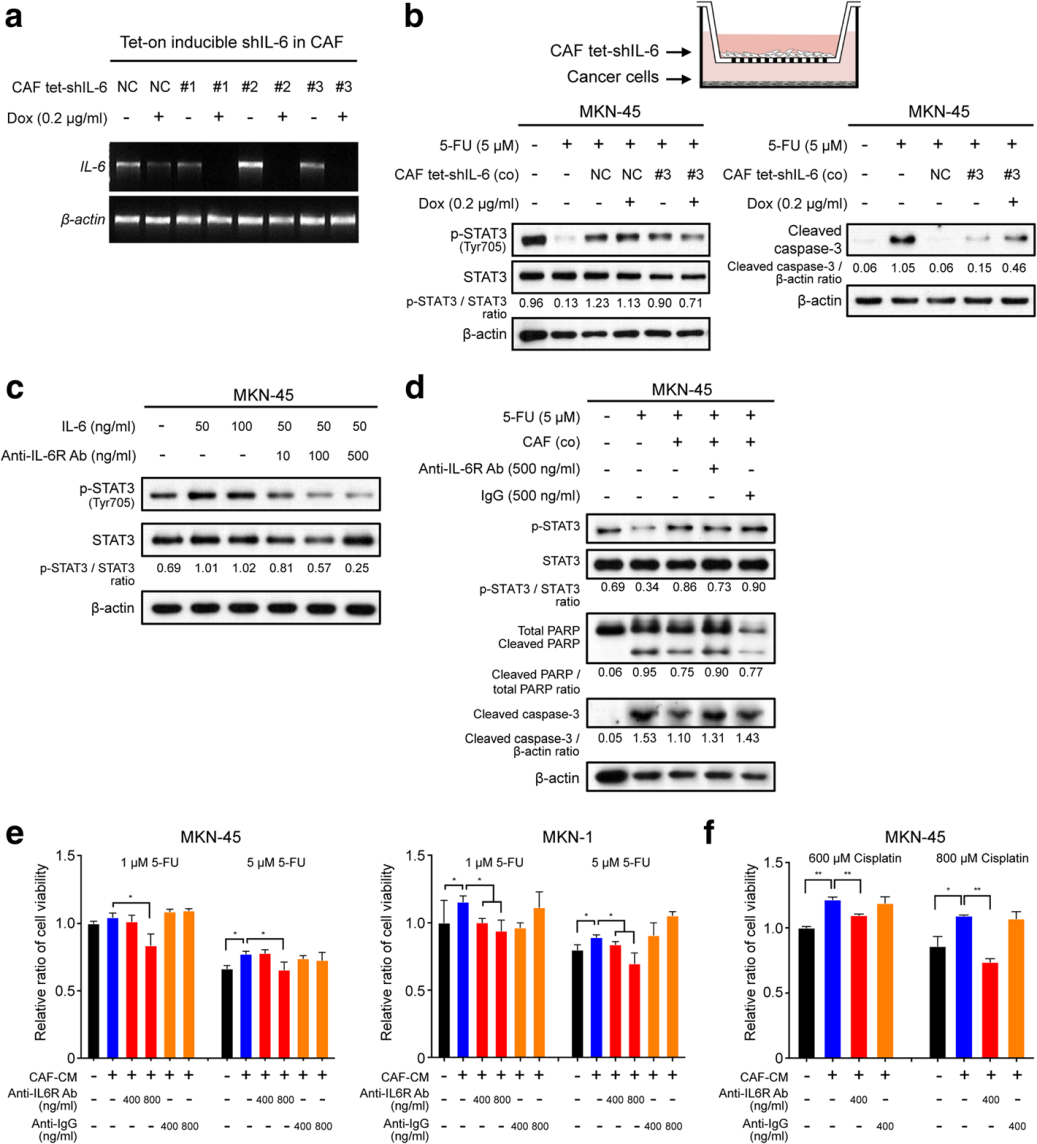
Suppressive effect of interleukin-6 (IL-6) inhibition on the cancer-associated fibroblast (CAF)-induced resistance to 5-fluorouracil (5-FU). **a** Reverse transcription (RT)-PCR analysis showing the expression of *IL6* and *ACTB* mRNA in CAFs transfected with three different tet-on inducible IL6 shRNAs vectors or a negative control vector [38]. Dox indicates doxycycline. **b** Schematic figure depicting the transwell co-culture system for tet-on IL6 shRNA-transfected CAFs and gastric cancer cells. Western blot analysis shows the expression of the apoptotic markers cleaved PARP, caspase-3, and phosphorylated STAT3 in the lysate of MKN-45 cell cultures in the lower chamber after doxycycline (0.2 μg/ml) treatment of CAFs transfected with the tet-on IL6 shRNA or negative control (NC) vector in the upper chamber. **c** Western blot analysis showing the expression of the indicated proteins in cells treated with human recombinant IL-6 combined with and without tocilizumab treatment. **d** Western blot analysis showing the expression of the indicated proteins in the lysates from MKN-45 and MKN-1 cells after 5-FU (5 μM) treatment with and without CAFs and subsequent treatment with tocilizumab (500 ng/ml) or negative control IgG (500 ng/ml). **e** Ez-cytox tests showing the relative ratio of the viability of MKN-45 and MKN-1 cells treated with 1 μM or 5 μM of 5-FU after the addition of tocilizumab (400 and 800 ng/ml) or control IgG (400 and 800 ng/ml). **f** Ez-cytox tests demonstrating the relative ratio of cell viability in MKN-45 cultures treated with 600 μM or 800 μM cisplatin after the addition of tocilizumab (400 ng/ml) or control IgG (400 ng/ml). The graphs show the mean (± SEM) ratios of cell viability. ^*^P < 0.05 and ^**^P < 0.001, according to Mann-Whitney test

To investigate the role of IL-6 in the development of chemotherapeutic resistance in GC cell lines, IL-6 in CAFs was silenced using a single-vector lentiviral doxycycline-inducible shRNA system. In the absence of doxycycline, IL-6 levels were similar in both the scramble shRNA-expressing cells and the shIL-6-expressing cells. In sharp contrast, the addition of doxycycline resulted in a significant downregulation of *IL6* mRNA expression (> 90% knockdown), as determined by RT-PCR analysis (Fig. 3a). Furthermore, MKN-45 cells that were transfected with the inducible vector containing either scramble shRNA or shIL-6 in the co-culture system were treated with 5-FU. After co-culturing with CAFs in which *IL6* was knocked down, the expression of p-STAT3 was downregulated. In contrast, the expression of cleaved caspase-3 increased in the co-culture system with the doxycycline-inducible tet-on shIL-6-treated CAFs upon 5-FU treatment (Fig. 3b).

We next examined the potential inhibitory activity of the monoclonal anti-IL-6R antibody tocilizumab on IL-6-induced STAT3 phosphorylation. As shown in Fig. 3c, phosphorylated STAT3 levels increased markedly in MKN-45 cells treated with recombinant IL-6. However, tocilizumab effectively abrogated its increase in a dose-dependent manner. To then investigate the effect of tocilizumab on CAF-mediated chemotherapeutic resistance in GC cells, we incubated tocilizumab with 5-FU in GC cells co-cultured with CAFs. Tocilizumab significantly alleviated the CAF-mediated chemoprotection in GC cells, as evidenced by the upregulation of the levels of cleaved caspase-3 and PARP, compared to the case for the treatment with a control IgG antibody. (Fig. 3d). CAF-induced JAK1 and STAT3 phosphorylation were also effectively reduced by the JAK1 inhibitor Ruxolitinib (Additional file 4: Figure S3). Cell viability assays revealed that co-treatment with CAF-CM significantly decreased the cytotoxic effect of chemotherapeutic agents on MNK-45 and MKN-1 cells. However, tocilizumab significantly decreased the chemoprotective capacity of CAF-CM in a dose-dependent manner (Fig. 3e, f). These data strongly suggest that the suppression of CAF-induced IL-6 secretion or pharmacologic inhibition of the IL-6 receptor counteracts the effect of CAF-induced chemotherapeutic resistance in GC cells.

### Tocilizumab reversed the effect of CAF-induced chemotherapeutic resistance in the xenograft mouse model of GC

**Fig. 4:**
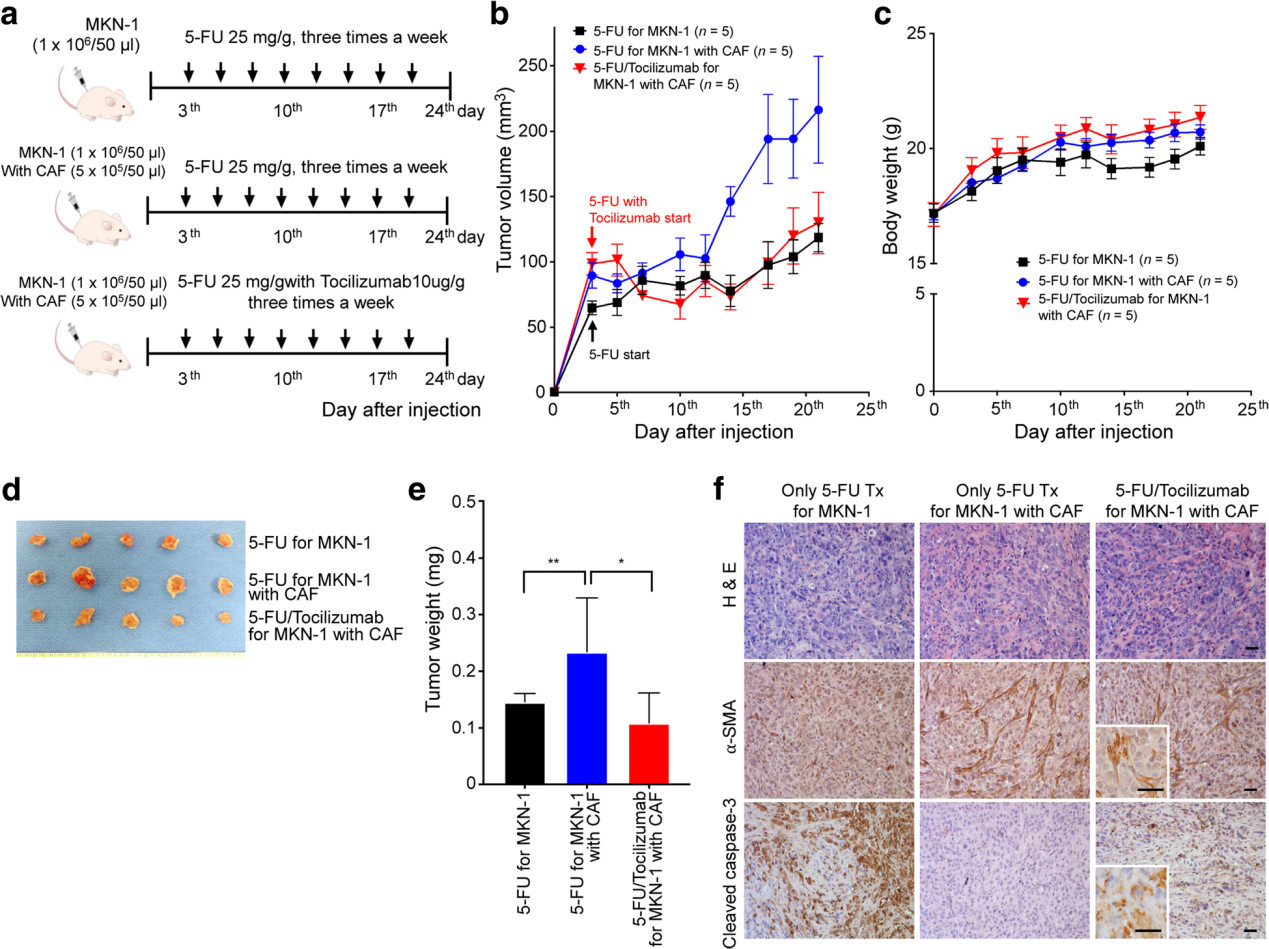
Effect of anti-interleukin-6 (IL-6) receptor monoclonal antibody on 5-fluorouracil (5-FU) treatment of mixed xenograft tumors derived from cancer-associated fibroblasts (CAFs). **a** The panels show representative images of the samples from each group. The arrows indicate the days on which the treatment was administered. **b** A line graph displaying the comparison of tumor growth among the in vivo xenograft tumors derived from MKN-1 cells alone (*n* = 5) and MKN-1 cells combined with CAFs (*n* = 5) after 5-FU treatments, and xenograft tumors derived from MKN-1 cells combined with CAFs (*n* = 5) treated simultaneously with 5-FU and tocilizumab. **c** A line graph showing the changes in body weight among the three groups of mice. **d** The photographs show the tumor-bearing mice before euthanasia and the harvested tumors. **e** The column graph comparing the harvested tumor weights among the three groups. The graphs show the mean (± SEM) tumor weights of the mice. ^*^P < 0.05 and ^**^P < 0.001, according to Mann-Whitney test. **f** Representative micrographs of the tumors harvested from the mice in the three groups; the tumor samples were analyzed by H&E staining and immunohistochemical staining for α-SMA and cleaved caspase-3. Scale bar = 100 μm

We investigated whether tocilizumab could attenuate CAF-induced chemotherapeutic resistance to 5-FU in an in vivo GC xenograft model. We prepared xenograft tumors derived from MKN-1 cells alone (*n* = 5) and MKN-1 cells mixed with CAFs (*n* = 10). On the third day following the subcutaneous injection of the cells, five mice with tumors derived from only MKN-1 cells and five mice with tumors derived from MKN-1 cells mixed with CAF were treated with 5-FU via intraperitoneal injection thrice a week for 3 weeks at a dose of 25 mg/g body weight. Five other mice with tumors derived from MKN-1 cells mixed with CAFs were also treated with tocilizumab (2 mg/ml) in the same manner as the 5-FU treatment (Fig. 4a). Consistent with previous findings, in mice treated with only 5-FU, CAF-mixed tumors showed a worse response to 5-FU than the tumors derived only from MKN-1 cells. When 5-FU treatment was supplemented with tocilizumab, the increase in the volumes of CAF-mixed tumors was suppressed in a manner similar to that in the tumors derived from only MKN-1 cells (Fig. 4b). There was no difference in the body weight of the mice among the three groups during treatment (Fig. 4c). In the mice treated with only 5-FU, the mean weight of the tumors harvested from the CAF-mixed xenografts was significantly greater than that of the xenografts derived from only MKN-1 cells (*P* = 0.018). The additional treatment with tocilizumab significantly reduced the tumor weight in CAF-mixed xenografts compared to those treated with only 5-FU (*P* = 0.047; Fig. 4d, e). Immunohistochemical staining revealed that the addition of tocilizumab to the 5-FU treatment for CAF-mixed tumors did not affect the accumulation of CAFs; however, it upregulated the expression of cleaved caspase-3 (Fig. 4f). Over all, the in vivo experiments revealed that tocilizumab treatment increased the sensitivity of the xenograft tumors containing CAFs to 5-FU through increased apoptosis without any observed side effects in the mice.

### Stroma-related genes including IL-6 in biopsied tissues might reduce the responsiveness to chemotherapy in GC

**Fig. 5:**
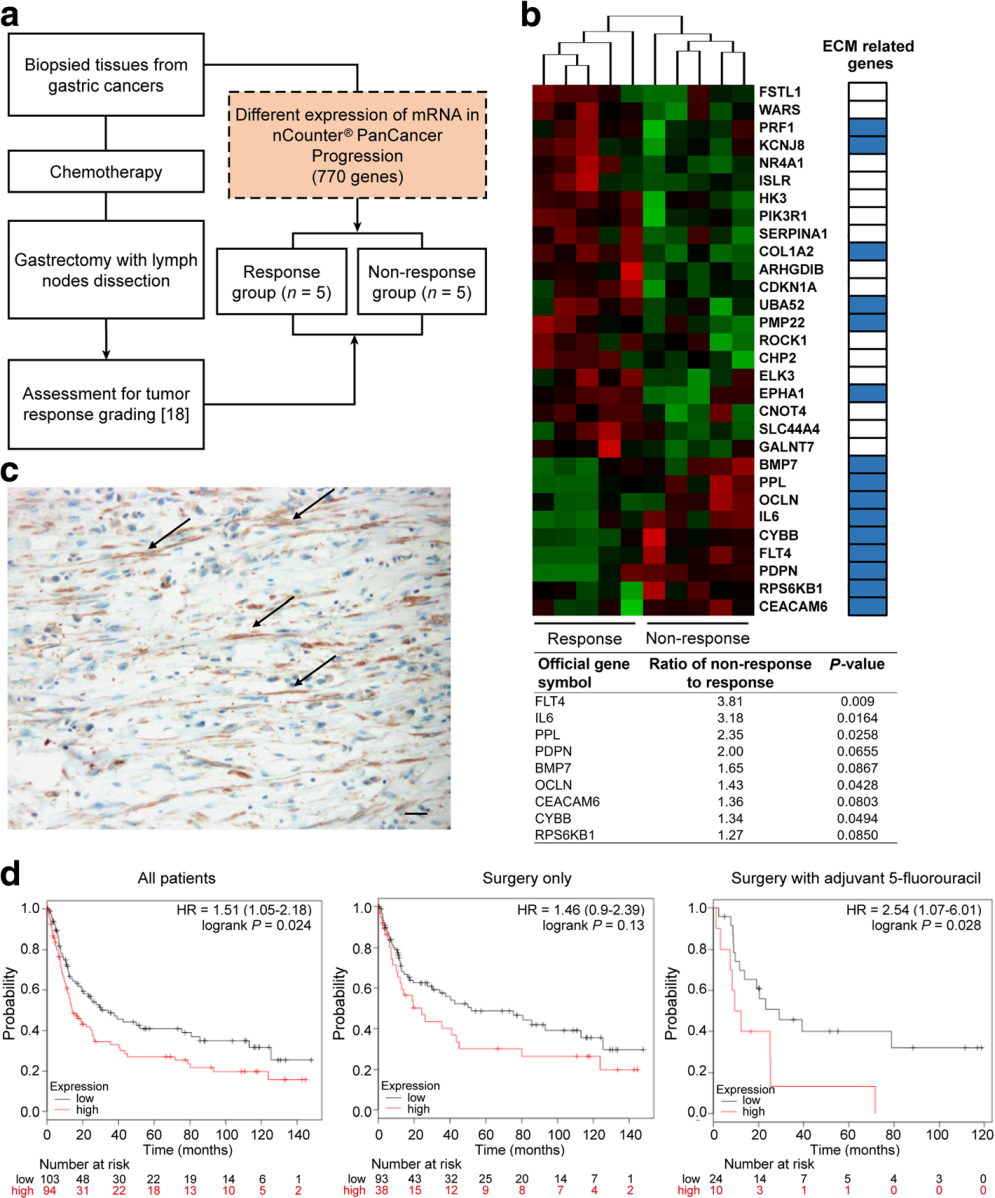
Gene expression pattern in pre-treatment biopsied tissues of patients who underwent preoperative chemotherapy. **a** Flow diagram presenting the study scheme for the comparison of gene expression patterns in the pre-treatment biopsied gastric cancer tissues between patients in the chemotherapy response and non-response groups. **b** Heatmap showing the top nine genes, including interleukin-6 (*IL-6*), usually those associated with the extracellular matrix layer, and the expression pattern of these genes appropriately clustered into the response and non-response patient groups. A list of upregulated genes is shown for the non-response group when gene expression was evaluated in biopsied tissues from primary tumors of pretreated gastric cancer patients **c** Representative micrograph of IL-6 immunohistochemical staining of gastric cancer tissues showing IL-6 expression specifically in the stromal cells (black arrows), but not in the cancer cells. Scale bar = 100 μm. **d** Disease-free survival assessed using the GSE15459 gastric cancer dataset at www.kmplot.com. The difference in survival relative to *IL6* mRNA expression was compared in each group involving all patients, i.e., those treated with surgery only and those treated with surgery and adjuvant chemotherapy, according to Kaplan-Meier survival analysis with log-rank test

The response to chemotherapy was evaluated using resected GC tissues from 10 patients with GC who underwent preoperative chemotherapy. Patients with a proportion of residual tumors of ≥50% were categorized into the non-response group according to a previous report that evaluated the chemo-response in colon cancer [21]. In total, five patients were categorized into the response group and five, into the non-response group (Fig. 5a). Gene expression profiling was performed on the biopsied GC tissues from the 10 patients treated with chemotherapy prior to surgery using an nCounter® PanCancer Progression Panel that included 770 genes involved in cancer progression processes, including angiogenesis, extracellular matrix (ECM) remodeling, epithelial-mesenchymal transition, and metastasis. Two-sample *t*-tests comparing the response and non-response groups identified 28 differentially regulated genes (FDR ≤ 10% and fold change ≥2.0; Fig. 5b). We selected the nine most highly upregulated genes in the non-response group for additional consideration. Interestingly, most of the genes represented a distinct ECM layer, and *IL6* showed the second highest fold change in expression (Fig. 5b). These results indicate that the expression of stroma-related genes in GC, including those for IL-6, may be associated with poor responsiveness to chemotherapy, since ECM-related proteins usually originate from tumor stromal cells.

A list of 105 genes (Additional file 5: Table S1) with the highest co-expression correlation with *IL6* in the TCGA GC dataset (Pearson r value > 0.4) were submitted to the Database for KEGG to perform gene ontology pathway enrichment analysis. The genes that positively correlated with IL-6 expression in GC were significantly enriched in the “stroma-related signature” such as cytokine-cytokine receptor interaction, chemokine signal transduction, ECM-receptor interaction, focal adhesion, pathway in cancer, Jak1-STAT signaling pathway, and others (Additional file 6: Table S2). Immunohistochemical staining was performed to analyze the expression pattern of IL-6 in the primary GC tissues. As shown in Fig. 5c, IL-6 was expressed in the cells with a fibroblast-like morphology. These results indicate that IL-6 in GC primarily originates from fibroblasts in the tumor stroma.

To validate our clinical findings in an independent dataset, *IL6* expression was analyzed using the GC dataset at www.kmplot.com. The desired Affymetrix ID for IL-6 was 205207_at. The survival curve for disease-free survival (DFS) after resection was plotted for 197 patients from the GSE15459 dataset. Patients with IL-6 upregulation had significantly worse DFS compared to those with IL-6 downregulation (*P* = 0.024, HR = 1.05–2.18). When the patients were stratified in accordance with adjuvant chemotherapy, among 131 patients not undergoing adjuvant chemotherapy, there was no difference in DFS between patients with a high expression of IL-6 and those with a low expression of IL-6 (*P* = 0.130, HR = 0.9–2.39). However, in 34 patients undergoing adjuvant chemotherapy, the patients with IL-6 upregulation showed significantly shorter DFS than those with IL-6 downregulation (*P* = 0.028, HR = 1.07–6.01; Fig. 5d). These clinical data support the conclusion that IL-6 in GC is primarily expressed in the stroma and IL-6 upregulation may be significantly associated with poor response to chemotherapy.

## Discussion

Our data indicate that IL-6 secreted by CAFs is critical for chemotherapeutic resistance in GC cells through the activation of the Jak1-STAT3 signaling pathway. The clinical data indicate that the expression of stroma-related genes, including IL-6, in biopsy specimens from patients treated with chemotherapy prior to surgery was significantly correlated with a poor response to chemotherapy in the GC patients. Finally, we showed that additional treatment with tocilizumab, a monoclonal antibody against the IL-6 receptor, in combination with chemotherapy, could serve as a suitable strategy to improve chemotherapeutic efficacy through the inhibition of the interaction between stromal and GC cells.

Our experimental data, including transcriptome analysis for paired NAFs and CAFs, demonstrated that CAFs secreted significantly higher amounts of IL-6 than NAFs, and thus, CAFs are more likely to contribute to chemotherapeutic resistance in GC cells than NAFs. Indeed, Lotti et al. have reported that IL-17A secretion is increased in fibroblasts isolated from colon cancer patients undergoing cytotoxic drug treatment regimens and subsequently causes chemotherapeutic resistance in colon cancer [22]. Another study has reported that cancer cells may activate CAFs in a paracrine manner, and as a result, several secretory factors such as CCL2, are upregulated, resulting in chemotherapeutic resistance in breast cancers [23]. In the current study, based on the analysis of multiple matched pairs of NAFs and CAFs, IL-6 was found to be significantly upregulated in CAFs, compared to NAFs, with respect to the levels of transcript and secreted protein (Fig. 2b–e). In addition, we found that the expression of IL-6 and the transcription factor NF-κB in CAFs was not altered by the co-culture with GC cells or by chemotherapy (Additional file 3: Figure S2b-d). Therefore, these data suggest that the irreversible activation of NAFs might drive IL-6 upregulation in CAFs. Recent comparative genomic analyses of CAFs and NAFs have identified genetic and epigenetic alterations in breast cancer, colon cancer, and ovarian cancer [24–26]. Moreover, Kalluri previously reported that epigenetic changes in NAFs irreversibly convert them into CAFs [27]; however, the exact mechanism underlying the increased IL-6 expression in CAFs derived from GC requires further investigation.

IL-6 is a multifunctional molecule involved in regulating immune and inflammatory responses [28]. However, recent studies have suggested that in various cancers, IL-6 may play a critical role in the communication between cancerous and non-cancerous cells within the tumor microenvironment. Some studies have reported that tumor-infiltrating immune cells such as M1 macrophages secrete high levels of IL-6 as an anti-tumor mediator, and that the increased accumulation of IL-6 is related to better prognosis in colorectal cancers [29, 30]. However, IL-6 from different sources, such as tumor cells, fibroblasts, and immune cells, is known to promote tumor growth, invasion, and anti-apoptotic potential in cancer cells [31, 32]. Previous studies have reported that autocrine secretion of IL-6 by cancer cells contributes to resistance to treatment [33–35]. However, through the comparison of IL-6 gene expression among various fibroblasts and GC cells, the current study shows that IL-6 within GC tumors is mainly produced by CAFs. The TCGA database also suggests that there is a co-expression of IL-6 in GC primary tumors with specific stroma-related genes such as those encoding ECM and focal adhesion molecules. Moreover, immunohistochemical staining of human GC tissues in the present study showed that IL-6 expression was localized to the stromal cells and not the cancer cells.

Previous studies have reported that IL-6 or IL-6 downstream signaling confers chemotherapeutic resistance by triggering the PI3K/Akt, MAPK/ERK, or Jak1/STAT3 signaling pathway in cancer cells [36–38]. PI3K/Akt or MAPK/ERK signal activation triggered by IL-6 has been reported to induce cancer cell proliferation through the upregulation of cyclin A1 in hepatoma, prostate cancer, and multiple myeloma [36, 39, 40]. However, IL-6-mediated STAT3 activation has frequently been suggested to be a protective mechanism in chemotherapy-induced cell death through the increased expression of anti-apoptotic proteins such as Bcl-2 or survivin in solid tumors such as breast cancer and prostate cancer [33, 41, 42]. In the present study, co-culture of GC cells with CAFs or CM from CAFs activated Jak-STAT3 signaling but not Akt signaling (Fig. 2f, g); however, treatment with human recombinant IL-6 activated both pathways (Fig. 2g). The downregulation of IL-6 in CAFs mediated by shRNA failed to promote chemotherapeutic resistance and did not increase the activation of STAT3 in the cancer cells co-cultured with CAFs (Fig. 3b). These findings suggest that the paracrine signaling of IL-6 derived from CAFs plays a crucial role in the development of chemotherapeutic resistance in GC, and that the IL-6/Jak1/STAT3 axis may serve as a suitable target to improve the therapeutic efficacy of chemotherapy.

Over the past few decades, the IL-6/Jak1/STAT3 axis has been widely targeted in the treatment of various inflammation-related diseases to alleviate patient symptoms [43, 44]. Tocilizumab, a humanized monoclonal anti-IL-6R antibody that is an FDA-approved drug for rheumatic arthritis and Crohn’s disease, competitively binds to both soluble and membrane-bound IL-6 receptors and blocks the intracellular IL-6 signaling pathway [45]. Since the role of IL-6 in cancer progression has been reported previously, it has also been investigated in experimental cancer models for various types of cancer, including oral, lung, ovarian, and breast cancers [14, 46–48]. However, only a phase II clinical trial including 18 patients with platinum-resistant ovarian cancer has yielded favorable results [49]. This clinical trial was based on experimental studies showing that tocilizumab inhibits the tumor growth and angiogenesis induced by IL-6 that normally leads to ovarian cancer. Nevertheless, only 1 of the 18 patients presented with a partial response, while the others showed stable disease or progression. This result implies that targeting only IL-6 may not be enough to achieve cytotoxic effects in cancer cells. Therefore, our approach used tocilizumab in combination with a traditional chemotherapeutic drug. In the current study, we hypothesize that tocilizumab could serve as a suitable targeting agent to increase the efficacy of chemotherapy, because we identified that in GC, CAFs, which are well-known contributors to chemotherapeutic resistance, were the major source of IL-6 in the tumor microenvironment. Our data from the animal studies showed that CAF-mixed xenograft tumors contained more fibroblasts and expressed IL-6 at higher levels within the tumors compared to the tumors not containing CAFs (Additional file 3: Figure S2a). Consequently, CAF-mixed tumors showed increased resistance to 5-FU, and the addition of tocilizumab to the treatment regimen facilitated a response and increased apoptosis in the cancer cells within the tumors, without having adverse effects in the mice (Fig. 4). These findings suggest that tocilizumab may be a suitable agent to overcome chemotherapeutic resistance in GC. However, because monoclonal antibodies such as tocilizumab may be harmful due to adverse effects such as gastrointestinal hemorrhage, thrombocytopenia, neutropenia, and upper respiratory tract infection [50], its clinical application should be more carefully considered.

Previous studies have reported that the expression of stroma-related genes in GC tissues is significantly correlated with poor responsiveness to chemotherapy [9, 10]. They studies indicate that stroma-related genes originate from the non-cancerous stromal component and not from the cancer cells. However, the authors of these studies analyzed gene expression in resected primary tumors, and their conclusions regarding drug responsiveness were dependent on tumor relapse after curative resection followed by adjuvant chemotherapy. They did not examine a direct correlation between gene expression patterns and drug responsiveness; thus, these previous results from gene expression analyses may not be useful in making decisions regarding neoadjuvant chemotherapy. In contrast, a major advantage of our study is the fact that we used biopsy specimens from GC patients who had been treated with chemotherapy prior to surgery. Our gene expression analysis revealed that upregulated genes, including IL-6, in pretreated biopsy tissues of pathologic non-responders after chemotherapy were primarily associated with the ECM, unlike the case for the responder group. Based on the TCGA data analysis, which shows that IL-6 in GC tissues was mainly co-expressed with stromal-related genes, we assumed that IL-6 expression in the biopsied tissues was higher within the stroma in the non-responsive group, compared to that in the responsive group. These findings suggest that gene expression analysis in pretreated biopsy tissues, using a Nanostring platform, may serve to guide the treatment of GC. However, our study has the following limitations. First, we analyzed only 10 GC patients who underwent gastrectomy for GC after chemotherapy. Neoadjuvant chemotherapy for GC is not a common modality in Eastern countries [51]; hence, it is difficult to identify patients who underwent preoperative chemotherapy. Second, the results using the Nanostring platform were not validated; however, this gene expression platform has previously generated high-quality, reproducible, results in quantity, even with small biopsied tissues of breast cancers [52, 53]. Future studies are required to determine whether this approach is applicable in clinical settings.

## Conclusions

**Fig. 6:**
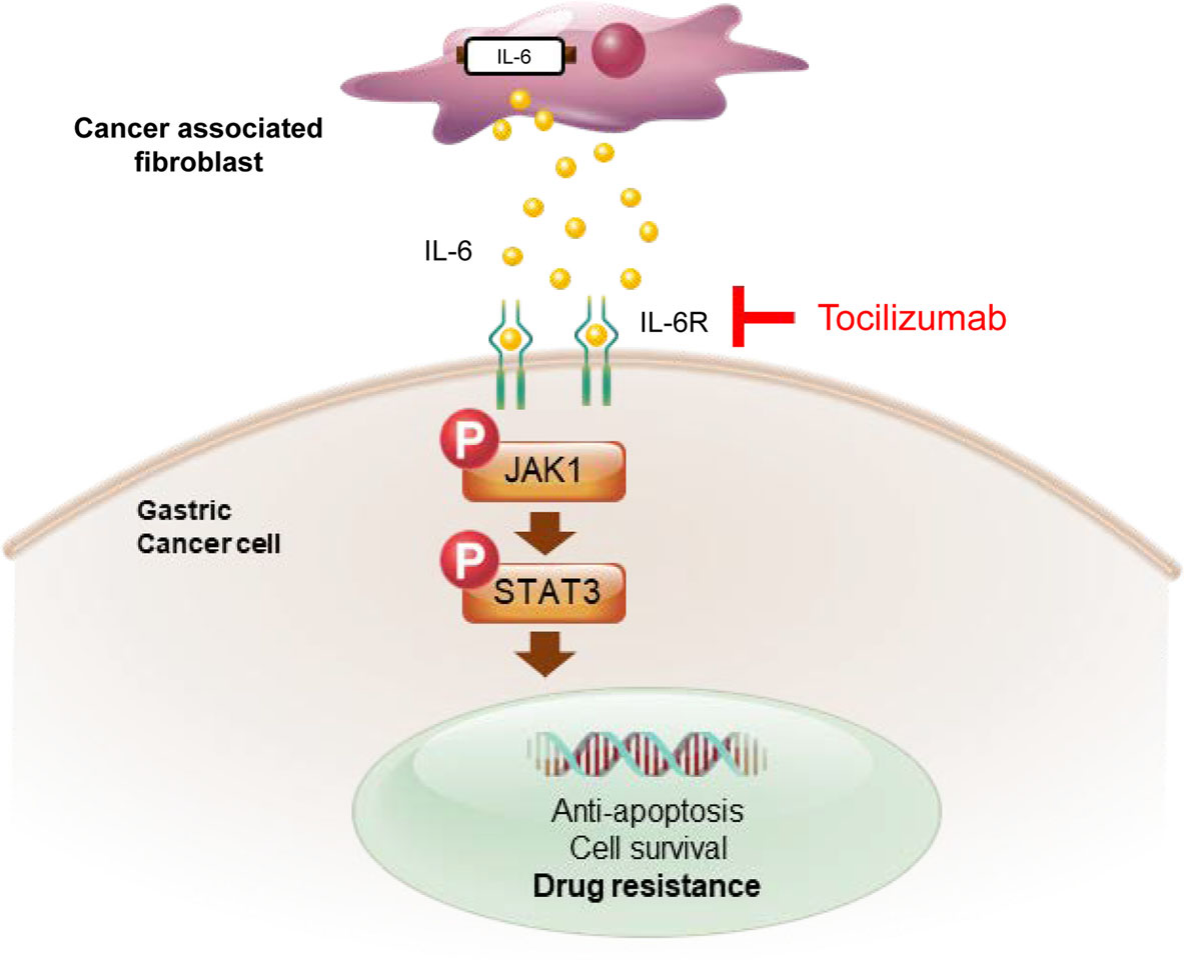
Schematic figure for present study. Cancer-associated fibroblast (CAF)-induced interleukin-6 (IL-6) activates the Jak1-STAT3 pathway in gastric cancer cells via paracrine signaling, which allows tumor cells to increasingly oppose apoptosis and increase their survival and resistance to chemotherapy. Tocilizumab, a humanized monoclonal anti-IL-6R antibody that is an FDA-approved drug, inhibits the CAF-induced activation of the Jak1-STAT3 signaling pathway in gastric cancer cells and consequently increases the efficacy of chemotherapeutic drugs

CAFs are the primary source of IL-6 in the tumor microenvironment of GC, and CAF-produced IL-6 activates the Jak1-STAT3 pathway in GC cells via paracrine signaling, resulting in the development of chemotherapeutic resistance. Therefore, the inactivation of the Jak1-STAT3 signaling axis with an anti-IL-6R monoclonal antibody effectively enhances responsiveness to chemotherapy (Fig. 6). Hence, we propose that blocking the interaction between cancer cells and CAFs by employing tocilizumab may have a clinical potential for GC treatment.

## Additional files

Additional file 1: Supplementary Materials and Methods. (DOCX 46 kb)

Additional file 2: **Figure S1. a** Cancer-associated fibroblasts (CAFs) culture-conditioned media used with MKN-45 and MKN-1 gastric cancer cells treated with cisplatin and the half maximal inhibitory concentration (IC_50_) sequentially measured. **b** A line graph comparing tumor growth among the in vivo xenograft tumors derived from MKN-45 cells alone (*n* = 6) and MKN-45 cells combined with CAFs (n = 6) after 5-fluorouracil (5-FU) treatment. The photographs show all the harvested tumors from the two groups of mice. (DOCX 208 kb)

Additional file 3: **Figure S2. a** Representative photographs displaying immunofluorescent staining for interleukin-6 (IL-6, in green) and DAPI staining of nuclei in the harvested xenograft tumors derived from MKN-1 cells only or MKN-1 cells mixed with cancer-associated fibroblasts (CAFs) after treatment with 5-fluorouracil (5-FU). **b** Results from quantitative PCR analysis showing the expression of *IL6* mRNA in CAF lysates with and without co-culture with gastric cancer cell lines MKN-45, MKN-28, and KATO-III. **c** ELISA showing the concentration of IL-6 in the CAF-conditioned media with and without co-culture with MKN-45 cells or treatment with 5-FU. **d** Western blot analysis demonstrating the expression of the indicated proteins in lysates of CAF cultures with and without co-culture with MKN-45 cells or 5-FU treatment. (DOCX 713 kb)

Additional file 4: **Figure S3. a** Western blot analysis demonstrating the expression of the indicated proteins in lysates from MKN-45 cells after 5-FU (5 μM) treatment with and without CAFs and subsequently treated with Ruxolitinib (500 nM/ml). (DOCX 187 kb)

Additional file 5: **Table S1.** The genes with highest co-expression correlation with IL-6 in TCGA gastric cancer dataset. (DOCX 24 kb)

Additional file 6: **Table S2.** The functional annotations of co-expressed genes with *IL6* in the TCGA gastric cancer dataset. (DOCX 19 kb)

## Abbreviations

5-FU: 5-fluorouracil
CAF: Cancer-associated fibroblast
CCL2: Chemokine (C-C motif) ligand 2
CM: Conditioned medium
DFS: Disease-free survival
ECM: Extracellular matrix
GC: Gastric cancer
gp130: Glycoprotein 130
IL-6: interleukin-6
Jak1-STAT3: Janus kinase 1-Signal transducer and activator of transcription 3
NAF: Normal-associated fibroblast
qRT-PCR: Quantitative reverse transcription PCR
RT-PCR: Reverse transcription PCR
sh: Short hairpin
SMA: Smooth muscle actin
TCGA: The Cancer Genome Atlas
