# Supplementary material for: Targeting interleukin-6 as a strategy to overcome stroma-induced resistance to chemotherapy in gastric cancer
Source: Mol Cancer. 2019 Mar 30;18:68. doi: 10.1186/s12943-019-0972-8 (PMC6441211; doi:10.1186/s12943-019-0972-8)
Supplement: Supplementary file 1 — Supplementary Materials and Methods. (DOCX 46 kb) [file 12943_2019_972_MOESM1_ESM.docx]

**Supplementary Materials and Methods**

**Cell lines and cell culture**

We purchased the GC cell lines MKN-1 (KCLB No. 80101) and MKN-45 (KCLB No. 80103) from the Korean Cell Line Bank (Seoul, Korea). These cells were cultured in RPMI-1640 medium supplemented with 10% fetal bovine serum (FBS; HyClone, Logan, UT, USA), 1% penicillin/streptomycin (Gibco, Detroit, MI, USA), and 1% amphotericin B (Sigma-Aldrich, St. Louis, MO, USA). The cells were incubated at 37 °C in a humidified incubator at 5% CO_2_.

**Isolation and culturing of fibroblasts**

Fibroblast isolation was approved by the institutional review board/ethics committee of Ajou University Hospital (AJIRB-BMR-GEN-14-376) and we got informed consent from all patients. Human stomach tumor specimens were obtained from patients that underwent tumor resection surgery at Ajou University Hospital (Suwon, Korea). The fibroblasts were isolated from GC tissues (CAF) and paired normal tissues (NAF) as previously described in the supplementary material and methods of Lee et al. (1). We used only ﬁbroblasts within six passages. In the current study, four paired NAF and CAF samples harvested from four different patients were used. The pathologic characteristics are described below.

| **Fibroblasts** | **Gender** | **Age**  **(year)** | **Tumor size (cm)** | **Depth of invasion** | **Location** | **Operation** | **WHO**  **classification** | **Lauren**  **classification** |
| --- | --- | --- | --- | --- | --- | --- | --- | --- |
| NAF1/CAF1 | Male | 57 | 15.0 | Serosa exposure | Whole | Total | Poorly tub | Mixed |
| NAF2/CAF2 | Female | 49 | 3.4 | Serosa exposure | Body | Total | Signet ring | Diffuse |
| NAF3/CAF3 | Male | 59 | 6.0 | Adjacent organ  Invasion | Antrum | Subtotal | Signet ring | Diffuse |
| NAF4/CAF4 | Male | 64 | 9.2 | Serosa exposure | Body | Total | Poorly tub | Mixed |
| **NAF**: normal gastric tissue-associated fibroblast; **CAF**: gastric cancer-associated fibroblast; WHO: world health organization | | | | | | | | |

**Co-culture with CAF or NAF**

MKN-1 and MKN-45 cells were seeded into the bottom chamber of 6-well transwell plates (Corning, Union City, CA, USA) at 1 × 10^5^ cells/well and then NAFs or CAFs were seeded onto the upper insert membrane (0.4-μm pore size). The chambers were then inserted into the well of the plate after 24 h. Two milliliters of DMEM (supplemented with 5% FBS, 1% penicillin, and streptomycin) was added to both the upper and the bottom chambers, allowing for interaction between the two cell types. The cells were incubated at 37 ℃ for 48 h.

**Western blot analysis**

The cells were washed with phosphate-buffered saline and lysed in lysis buffer. Lysates were incubated on ice for 20 min and centrifuged at 13,000 rpm for 20 min at 4 °C. Protein concentrations were determined using the Bradford assay (Bio-Rad, Richmond, CA, USA). Samples with equalized protein concentrations were subjected to SDS-PAGE and electroblotted onto polyvinylidene difluoride membranes (Millipore, Billerica, MA, USA). The immunoblots were blocked by incubation in 5% skimmed milk, 25 mM Tris-HCl (pH 8.0), 150 mM NaCl, and 0.1% Tween® 20 for 1 h at room temperature. The membranes were incubated with the following primary antibodies, anti-STAT3 (1:1,000; #9139, Cell Signaling Technology, Danvers, MA, USA), anti-phosphorylated-STAT3 (1:1,000; #9145, Cell Signaling Technology), anti-phosphorylated-Jak1 (1:1,000; #3331, Cell Signaling Technology), anti-phosphorylated-p44/42 MAPK (1:1,000; #4370, Cell Signaling Technology), anti-cleaved PARP (1:1,000; #9542, Cell Signaling Technology), anti-cleaved caspase-3 (1:1,000; #9664, Cell Signaling Technology), and anti-β-actin (1:5,000; #sc-47778, Santa Cruz Biotechnology, Dallas, TX, USA). This was followed by incubation with the corresponding HRP-conjugated secondary antibodies (1:5,000; #AbC-5001/#AbC-5003, Abclon, Seoul, Korea). Proteins were detected using an enhanced chemiluminescence kit (Abclon).

**Secretome analysis**

To identify upregulated secretory factors in the culture supernatants of co-cultured MKN-45 cells with CAF relative to that in culture supernatants of MKN-45 cells without CAF co-culturing, we performed secretome analysis using a Proteome Profiler Human Cytokine Array Kit (R&D Systems Inc., Minneapolis, MN). Conditioned medium (CM) from MKN-45 cells alone or co-cultured with CAFs in serum-free medium were collected after 48 h and incubated with arrays containing 36 human cytokine specific antibodies according to the manufacturer’s instructions.

**Transcriptome analysis and quantitative real-time PCR (qRT-PCR)**

Total RNA was isolated using a Total RNA Isolation Kit (Qiagen, Hilden, Germany) in accordance with the manufacturer’s instructions. Complementary DNA (cDNA) was generated using 1 μg total RNA per sample as template and a cDNA Synthesis Master Mix Kit (GenDEPOT, Barker, TX, USA). To investigate the differences in gene expression patterns between a matched pair of NAFs and CAFs, we performed transcriptome analysis using the NextSeq 500 RNA sequencing platform (Illumina, San Diego, CA, USA). Gene expression data from the NAFs and CAFs have been deposited under Gene Expression Omnibus accession number GSE 112350. To validate the results of transcriptome analysis, qRT-PCR was performed using a Bio-rad RealTime PCR System (Bio-rad) and the four matched pairs of NAFs and CAFs.

Primers:

| **Gene symbol** | **Primer sequence** |
| --- | --- |
| Human_*ACTA2*_For | 5´-CCGACCGAATGCAGAAGGA-3´ |
| Human_*ACTA2*_Rev | 5´-ACAGAGTATTTGCGCTCCGAA-3´ |
| Human_*IL6*_For | 5´-GGCACTGGCAGAAAACAACC-3´ |
| Human_*IL6*_Rev | 5´-GCAAGTCTCCTCATTGAATCC-3´ |
| Human_*IL12A*_For | 5´-CCTTCACCACTCCCAAAAC-3´ |
| Human_*IL12A*_Rev | 5´-TGTCTGGCCTTCTGGAGCAT-3´ |
| Human_*IL24*_For | 5´-CAGGCGGTTTCTGCTATTCC-3´ |
| Human_*IL24*_Rev | 5´-GGCGTGAAGTGTCCAGTGAA-3´ |
| Human_*GAPDH*_For | 5´- GAAGGTGAAGGTCGGAGT -3´ |
| Human_*GAPDH*_Rev | 5´-GAAGATGGTGATGGGATTTC-3´ |

The qRT-PCR was carried out using 1 ug RNA equivalent of cDNA, iTaq™ universal SYBR® Green Supermix (BIO-RAD), and 100 pM 1 gene-specific primers. The PCR cycling conditions were as follows: 95 ℃ for 30 min followed by sequential cycles of 95 ℃ for 10 s and 60 ℃ for 30 s with a melting step. The intensity of the fluorescent dye was determined and the expression level of each mRNA was normalized to that of *GAPDH*.

**Reverse transcriptase polymerase chain reaction (RT-PCR)**

Total RNA extracted from monocytes, gastric cancer cells, and fibroblasts was converted to cDNA using 1 μg of RNA from each cell type as template in a final volume of 20 μl. The cDNA mixture was subjected to 40 cycles of PCR amplification using the following cycling conditions: denaturation at 95 ℃ for 30 s, annealing at 60 ℃ for 30 s, and extension at 72 ℃ for 1 min. The PCR products were visualized by agarose gel electrophoresis.

Primers:

| **Gene symbol** | **Primer sequence** |
| --- | --- |
| Human_*IL6*_For | 5´-TCCACAAGCGCCTTCGGTCC-3´ |
| Human_*IL6*_Rev | 5´-TTGCCGAAGAGCCCTCAGGCT-3´ |
| Human_*mIL6*_For | 5´-CATTGCCATTGTTCTGAGGTTC-3´ |
| Human_*sIL6R*_For | 5´-GCGACAAGCCTCCCAGGTTC-3´ |
| Human_*IL6R*_Rev | 5´-GTGCCACCCAGCCAGCTATC-3´ |
| Human_*gp130*_For | 5´-GGTACGAATGGCAGCATACA-3´ |
| Human_*gp130*_Rev | 5´-CTGGACTGGATTCATGCTGA-3´ |
| Human_*β-actin*_For | 5´-AGGCATCCTCACCCTGAAGTA-3´ |
| Human_*β-actin*_Rev | 5´-CACACGCAGCTCATTGTAGA-3´ |

**Cell viability assay**

Cells in each group were seeded into 96-well culture plates (10,000–15,000 cells/well) and incubated for 72 h at 37 ℃ with 5% CO_2_ and 95% O_2_. Thereafter, Ez-Cytox reagent was added to each well and incubated for 1–2 h at 37 ℃. The cell viability/proliferation assay was performed using an EZ-Cytox Assay Kit (Ez-Cytox cell viability, proliferation assay kit; Cat No. Ez-1000, DoGen, DaeillLab, Korea). The absorbance of the samples was measured using a microplate reader at 450 nm. Experiments were performed in triplicates.

**Immunohistochemical staining**

Formalin-fixed paraffin-embedded human or xenograft tumors were sectioned, affixed onto microscope slides, deparaffinized with xylene, hydrated using a diluted alcohol series, and immersed in 0.3% H_2_O_2_ in methanol to quench endogenous peroxidase activity. Sections were then treated with citrate buffer (10 mM, pH 6.0) for antigen retrieval. To reduce non-specific staining, each section was treated with 20% Aquablock (Abcam, Cambridge, UK) in TBS with 0.2% Tween® 20 (TBST) for 30 min. Sections were then incubated overnight at 4 °C with the following mouse/rabbit-derived primary antibodies: monoclonal anti-IL-6 (1:100; #ab9324, Abcam, Cambridge, UK), monoclonal anti-cleaved caspase-3 (1:100; #9664, Cell Signaling Technology), polyclonal anti-alpha-SMA (1:100; #RB-9010, Thermo Fisher Scientific, Waltham, MA, USA) in Antibody Diluent Solution (GBI Labs, Bothell, WA, USA). The following day, after three successive rinses with a washing buffer, the sections were incubated with polyclonal anti-mouse/rabbit secondary antibodies (1:5,000; #AbC-5001/#AbC-5003, Abclon) for 60 min at room temperature. The chromogen used was 3,3´-diaminobenzidine (Thermo Fisher Scientific). Sections were counterstained with Harris hematoxylin.

**Immunofluorescence assay**

Paraffin-embedded sections from MKN-1 and MKN-45 xenograft tumors were deparaffinized with xylene, hydrated using a diluted alcohol series, and immersed in 0.3% H_2_O_2_ in methanol to quench endogenous peroxidase activity. Sections were then treated with citrate buffer (10 mM, pH 6.0) for antigen retrieval. To reduce non-specific staining, each section was treated with 20% Aquablock (Abcam) in TBST for 30 min. Sections were then incubated overnight at 4 °C with the following mouse-derived primary antibodies: monoclonal anti-IL-6 (1:100; #ab9324, Abcam) in Antibody Diluent Solution (GBI Labs). The following day, samples were washed with TBST and incubated with Alexa 488 goat anti-mouse IgG and Cy3-labeled goat anti-rabbit IgG secondary antibodies (1:100-200; #115-545-146/#111-165-144, Jackson ImmunoResearch, PA, USA) for 1 h. Nuclei were stained with DAPI and sections were mounted with Vectashield mounting medium with DAPI for fluorescence detection (#H-1200, Vector lab, Burlingame, CA, USA).

**Generation of inducible short hairpin (sh) RNA for IL-6 (*shIL6*)**

To generate shRNA-expressing plasmids, double-stranded oligos encoding the desired shRNA were cloned into the single-vector inducible shRNA construct pLKO-Tet-On. Target cell lines were transduced with lentiviral inducible shRNA followed by selection in 1 μg/ml puromycin-containing growth medium until no non-transduced cells remained. The double-stranded oligos were inserted between the Age1/EcoR1 restriction sites of the pLKO-Tet-On vector for generation of the inducible RNAi. The sequences including the follow:

| **Oligos names** | **Oligos sequence** |
| --- | --- |
| Control #SHC001 | pLKO.1-puro empty vector control plasmid DNA |
| *IL6* #1 TRCN0000372667: top | 5´-CCGGATGAGCGTTAGGACACTATTTCTCGAGAAATAGTGTCCTAACGCTCATTTTTT-3´ |
| *IL6* #1 TRCN0000372667: bottom | 5´-AATTAAAAAATGAGCGTTAGGACACTATTTCTCGAGAAATAGTGTCCTAACGCTCAT-3´ |
| *IL6* #2 TRCN0000372668: top | 5´-CCGGATGTGAAGCTGAGTTAATTTACTCGAGTAAATTAACTCAGCTTCACATTTTTT-3´ |
| *IL6* #2 TRCN0000372668: bottom | 5´–AATTAAAAAATGTGAAGCTGAGTTAATTTACTCGAGTAAATTAACTCAGCTTCACAT-3´ |
| *IL6* #3 TRCN0000372669: top | 5´-CCGGCAAAGAATCTAGATGCAATAACTCGAGTTATTGCATCTAGATTCTTTGTTTTT-3´ |
| *IL6* #3 TRCN0000372669: bottom | 5´-AATTAAAAACAAAGAATCTAGATGCAATAACTCGAGTTATTGCATCTAGATTCTTTG-3´ |

Doxycyline (0.2 µg/ml) was added to the media to determine the infection efficiency and for maintenance unless otherwise noted. Non-transduced cells died after 4 d of selection. Puromycin-resistant cells were pooled and analyzed as populations rather than as clones.

**Animal model study**

Animal care and handling procedures were performed in accordance with the Ajou University School of Medicine Institutional Animal Care and Use Committee guidelines and all animal experiments were approved by the Animal Research Committee of the institution (IACUC protocol 2015-0069). The animal model was established using 6- to 8-week-old female athymic nude mice (Orient Bio, Gyonggi-Do, Korea) weighing 16 to 18 g. To establish xenograft tumors, the cells were suspended in 50 μl Matrigel mixed with PBS. In total, 1 × 10^6^ tumor cells (MKN-1 or MKN-45) with or without 1 × 10^5^ fibroblasts were subcutaneously implanted into the hind flank of BALB/c-*nu* nude mice (Orient Bio). Three or 5 days after cell transplantation, when the tumor size was more than 50mm^3^, mice were treated intraperitoneally with 5-FU (25 mg/g body weight) three times a week for 3 wk. To investigate the effect of IL-6 receptor inhibition, we injected tocilizumab (10ug/g) into the peritoneum of the mice. Tumor volume and body weight were monitored throughout the study period. In all experiments, tumor dimensions were measured using calipers, and tumor volume was calculated using the following formula: tumor volume (mm^3^) = (*a* x *b*^2^)/^2^, where *a* = length in mm and *b* = width in mm. After euthanizing the mice at the end of the study period, all tumors were harvested for measurement of the tumor weight and for immunohistochemical staining.

**The Cancer Genome Atlas (TCGA) Data**

We analyzed the TCGA Stomach Adenocarcinoma dataset using the cBioportal tool (<http://www.cbioportal.org)>. This dataset includes mRNA expression data from 295 primary GC patients. Analyzing the genes positively co-expressed with IL-6, we selected genes that had a Pearson correlation coefficient score of 0.4 and more with *IL6* mRNA expression. The functional annotation for the selected genes was analyzed using DAVID Bioinformatics Resources 6.8 for KEGG and Gene Ontology (GO) pathway enrichment analysis.

**Gene expression analysis in biopsied GC tissues**

To assess differential gene expression in biopsy samples in response to chemotherapy, 10 GC patients treated preoperatively with 5-FU-based chemotherapies followed by surgical resection for various reasons, were included in this study.

Patient list:

| **No** | **Age** | **Gender** | **Histology in biopsied tissues (WHO)** | **Pre-treatment clinical stage** | **Regiment for chemotherapy** | **Cycle** | **Time to operation**  **from chemotherapy**  **(Month)** | **Pathologic Response to chemotherapy** |
| --- | --- | --- | --- | --- | --- | --- | --- | --- |
| 1 | 59 | male | Poorly | T2N2M1 | S-1+CDDP | 3 cycles | 3 | Response |
| 2 | 62 | female | Poorly | T3N3M1 | TCF | 3 cycles | 10 | Non-response |
| 3 | 54 | male | Moderately | T4bN2M1 | DCF | 14 cycles | 12 | Response |
| 4 | 51 | female | Poorly cohesive | T4bN2M0 | FLFIRI | 9 cycles | 11 | Non-response |
| 5 | 77 | male | Poorly cohesive | T2bN3M1 | FOLFOX | 12 cycles | 7 | Non-response |
| 6 | 56 | male | Moderately | T3N1M1 | FOLFOX | 9 cycles | 5 | Non-response |
| 7 | 45 | male | Moderately | T4aN1M0 | S-1+CDDP | 6 cycles | 6 | Response |
| 8 | 53 | male | Moderately | T4aN2M0 | DOS | 3 cycles | 3 | Response |
| 9 | 51 | male | Moderately | T4N3M1 | S-1+CDDP | 2 cycles | 14 | Non-response |
| 10 | 72 | female | Moderately | T4bN3M1 | FOLFOX | 11 cycles | 6 | Response |

This study was approved by the institutional review board/ethics committee of Ajou University Hospital (AJIRB-BMR-KSP-15-432). The harvested tissue samples were formalin-fixed paraffin-embedded (FFPE), which included biopsied tissues and primary gastric tumors surgically resected from patients that received chemotherapy prior to surgery. All hematoxylin and eosin-stained sections were reviewed by experienced gastrointestinal pathologists to confirm the inclusion of cancer cells in the biopsied tissues and to be assigned a grade of responsiveness to chemotherapy in the primary GC tissues in accordance with a previously reported grading system for gastrointestinal cancers (2).

To analyze mRNA expression in the biopsied tissues, total RNA was extracted from 5–6 tissue blocks and median yield of RNA concentration was 36.78 ng/ul (range: 12.94 to 150.92) and purity based on A_260/280_ ratio was 1.718 (range: 1.006 to 2.393). Gene expression profiling was performed on the samples using nCounter® PanCancer Progression Panel (Nanostring Technologies, Seattle, WA, USA).

**Statistical analysis**

All experiments were performed independently in triplicate. Results are shown a means ± standard errors (SE). To compare the means between two groups, datasets were analyzed using an unpaired or paired *t*-test for normally distributed data. Otherwise, Mann-Whitney U test or Wilcoxon test were used. For comparison of means among three or more groups, one-way analysis of variance (ANOVA) followed by post hoc Turkey’s test analysis was used. Statistical analysis was performed using IBM SPSS statistics software (version 21 for Mac OS X, IBM, Armonk, NY, USA) and GraphPad Prism software (version 6.0 for Mac OS X, GraphPad, La Jolla, CA, USA) and the results were considered statistically significant at *P* < 0.05* or *P* < 0.001*.

**References:**

1. Lee D, Ham IH, Son SY, Han SU, Kim YB, Hur H. Intratumor stromal proportion predicts aggressive phenotype of gastric signet ring cell carcinomas. Gastric Cancer 2017;20:591-601.

2. Rodel C, Martus P, Papadoupolos T, Fuzesi L, Klimpfinger M, Fietkau R, et al. Prognostic significance of tumor regression after preoperative chemoradiotherapy for rectal cancer. J Clin Oncol 2005;23:8688-96.
