# Supplementary material for: Targeting interleukin-6 as a strategy to overcome stroma-induced resistance to chemotherapy in gastric cancer
Source: Mol Cancer. 2019 Mar 30;18:68. doi: 10.1186/s12943-019-0972-8 (PMC6441211; doi:10.1186/s12943-019-0972-8)
Supplement: Supplementary file 2 — Figure S1. a Cancer-associated fibroblasts (CAFs) culture-conditioned media used with MKN-45 and MKN-1 gastric cancer cells treated with cisplatin and the half maximal inhibitory concentration (IC50) sequentially measured. b A line graph comparing tumor growth among the in vivo xenograft tumors derived from MKN-45 cells alone (n = 6) and MKN-45 cells combined with CAFs (n = 6) after 5-fluorouracil (5-FU) treatment. The photographs show all the harvested tumors from the two groups of mice. (DOCX 208 kb) [file 12943_2019_972_MOESM2_ESM.docx]

**
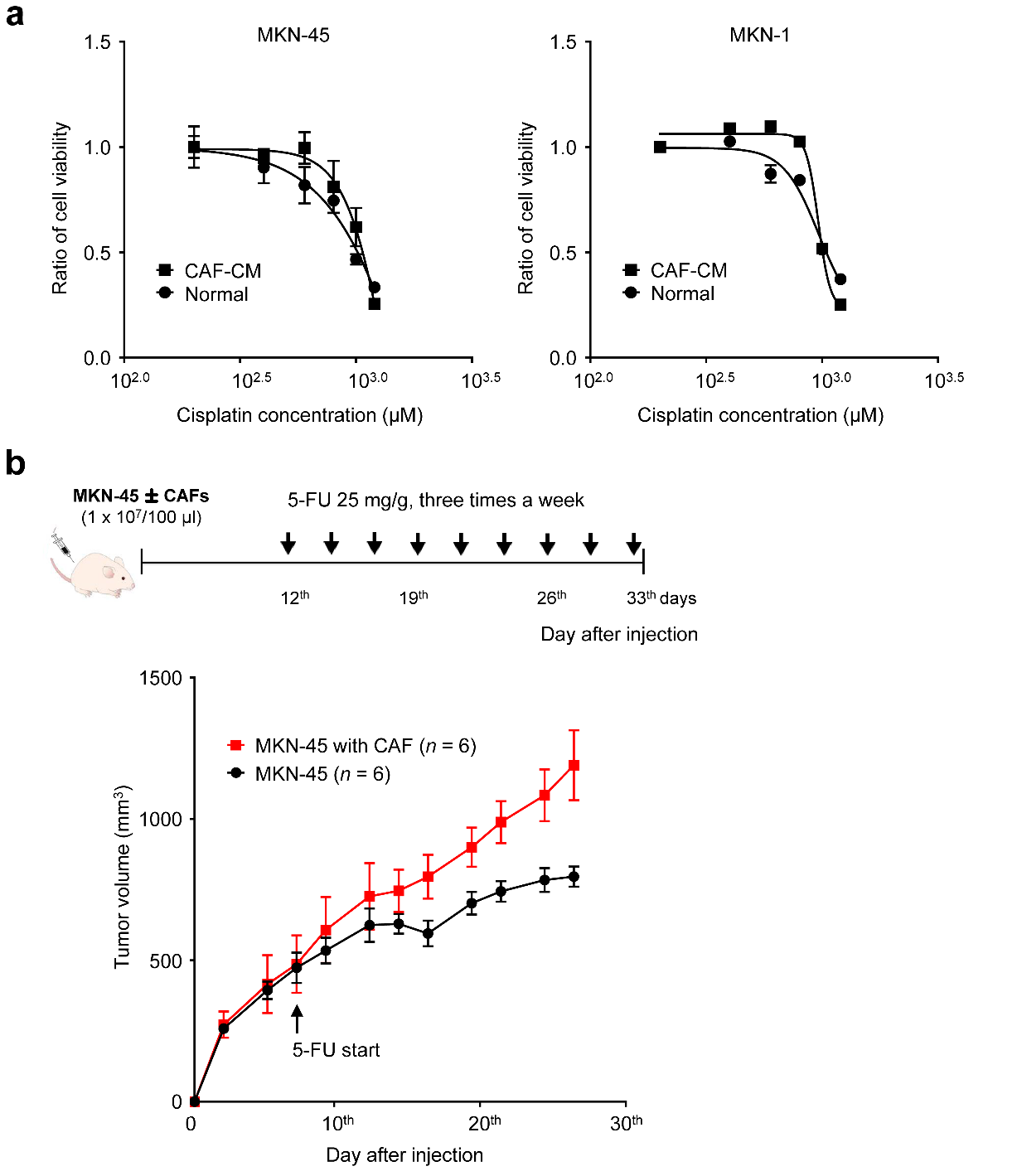
**

**Figure S1.**

**a** Cancer-associated fibroblasts (CAFs) culture-conditioned media used with MKN-45 and MKN-1 gastric cancer cells treated with cisplatin and the half maximal inhibitory concentration (IC_50_) sequentially measured. **b** A line graph comparing tumor growth among the *in vivo* xenograft tumors derived from MKN-45 cells alone (n = 6) and MKN-45 cells combined with CAFs (n = 6) after 5-fluorouracil (5-FU) treatment. The photographs show all the harvested tumors from the two groups of mice.
