# Supplementary material for: Targeting interleukin-6 as a strategy to overcome stroma-induced resistance to chemotherapy in gastric cancer
Source: Mol Cancer. 2019 Mar 30;18:68. doi: 10.1186/s12943-019-0972-8 (PMC6441211; doi:10.1186/s12943-019-0972-8)
Supplement: Supplementary file 3 — Figure S2. a Representative photographs displaying immunofluorescent staining for interleukin-6 (IL-6, in green) and DAPI staining of nuclei in the harvested xenograft tumors derived from MKN-1 cells only or MKN-1 cells mixed with cancer-associated fibroblasts (CAFs) after treatment with 5-fluorouracil (5-FU). b Results from quantitative PCR analysis showing the expression of IL6 mRNA in CAF lysates with and without co-culture with gastric cancer cell lines MKN-45, MKN-28, and KATO-III. c ELISA showing the concentration of IL-6 in the CAF-conditioned media with and without co-culture with MKN-45 cells or treatment with 5-FU. d Western blot analysis demonstrating the expression of the indicated proteins in lysates of CAF cultures with and without co-culture with MKN-45 cells or 5-FU treatment. (DOCX 713 kb) [file 12943_2019_972_MOESM3_ESM.docx]

**
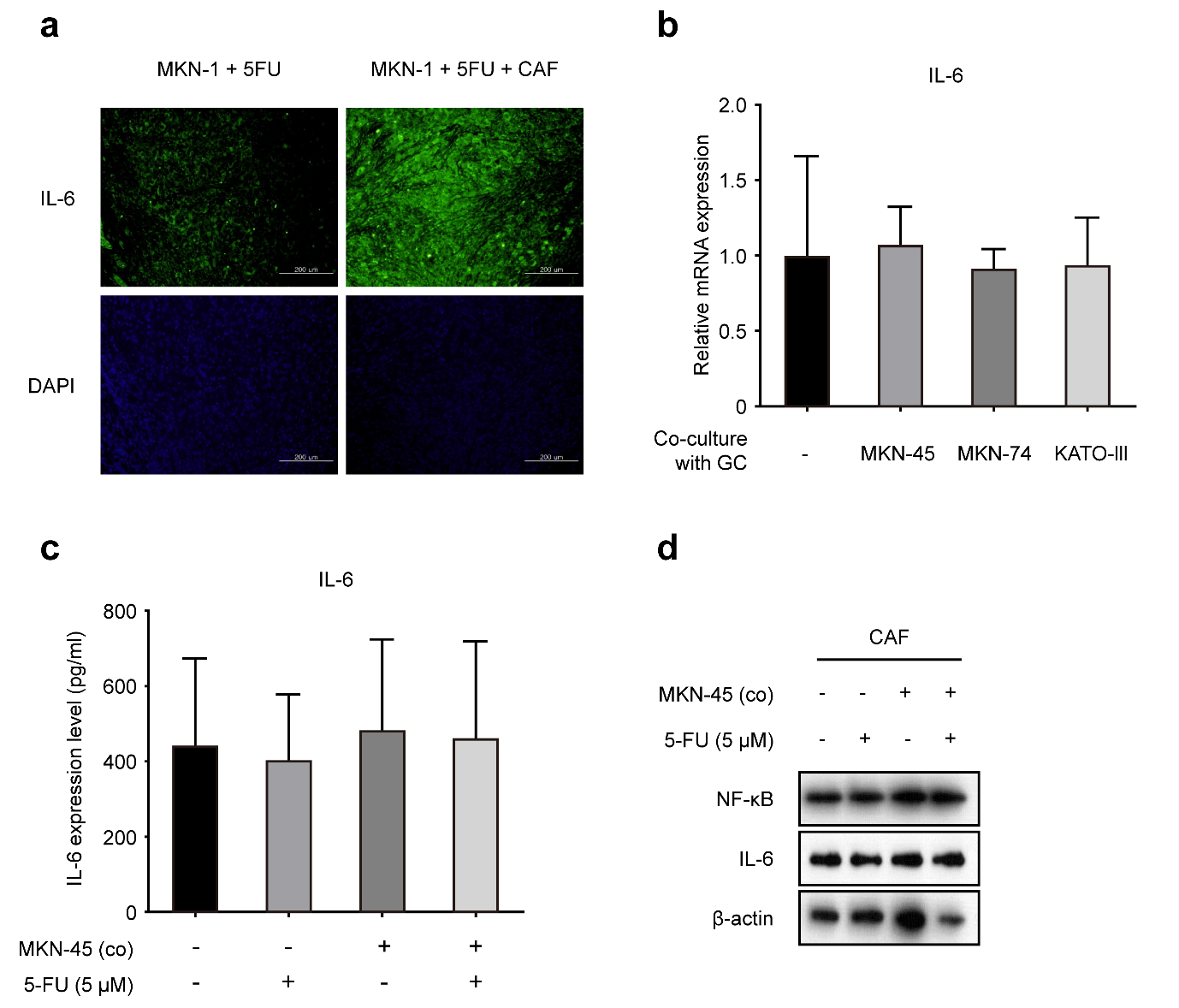
**

**Figure S2.**

**a** Representative photographs displaying immunofluorescent staining for interleukin-6 (IL-6, in green) and DAPI staining of nuclei in the harvested xenograft tumors derived from MKN-1 cells only or MKN-1 cells mixed with cancer-associated fibroblasts (CAFs) after treatment with 5-fluorouracil (5-FU). **b** Results from quantitative PCR analysis showing the expression of *IL6* mRNA in CAF lysates with and without co-culture with gastric cancer cell lines MKN-45, MKN-28, and KATO-III. **c** ELISA showing the concentration of IL-6 in the CAF-conditioned media with and without co-culture with MKN-45 cells or treatment with 5-FU. **d** Western blot analysis demonstrating the expression of the indicated proteins in lysates of CAF cultures with and without co-culture with MKN-45 cells or 5-FU treatment.
