# Supplementary material for: Targeting interleukin-6 as a strategy to overcome stroma-induced resistance to chemotherapy in gastric cancer
Source: Mol Cancer. 2019 Mar 30;18:68. doi: 10.1186/s12943-019-0972-8 (PMC6441211; doi:10.1186/s12943-019-0972-8)
Supplement: Supplementary file 4 — Figure S3. a Western blot analysis demonstrating the expression of the indicated proteins in lysates from MKN-45 cells after 5-FU (5 μM) treatment with and without CAFs and subsequently treated with Ruxolitinib (500 nM/ml). (DOCX 187 kb) [file 12943_2019_972_MOESM4_ESM.docx]

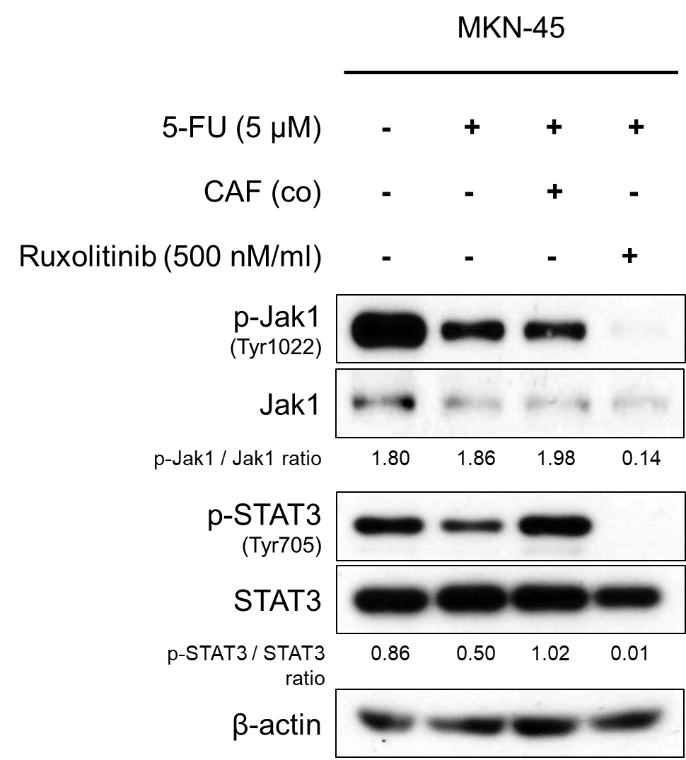


**Figure S3.**

**a** Western blot analysis demonstrating the expression of the indicated proteins in lysates from MKN-45 cells after 5-FU (5 μM) treatment with and without CAFs and subsequently treated with Ruxolitinib (500 nM/ml).
