# Supplementary material for: Targeting interleukin-6 as a strategy to overcome stroma-induced resistance to chemotherapy in gastric cancer
Source: Mol Cancer. 2019 Mar 30;18:68. doi: 10.1186/s12943-019-0972-8 (PMC6441211; doi:10.1186/s12943-019-0972-8)
Supplement: Supplementary file 5 — Table S1. The genes with highest co-expression correlation with IL-6 in TCGA gastric cancer dataset. (DOCX 24 kb) [file 12943_2019_972_MOESM5_ESM.docx]

**Table S1** The genes with highest co-expression correlation with IL-6 in TCGA gastric cancer dataset

| Gene symbol | Cytoband | Pearson score | Gene symbol | Cytoband | Pearson score |
| --- | --- | --- | --- | --- | --- |
| *LOC541472* | 7p21 | 0.94 | *FAM132B* | 2q37.3 | 0.47 |
| *PROK2* | 3p13 | 0.69 | *CHSY3* | 5q23.3 | 0.47 |
| *CSF3* | 17q11.2-q12 | 0.62 | *LOX* | 5q23.2 | 0.46 |
| *IL11* | 19q13.3-q13.4 | 0.62 | *LBP* | 20q11.23 | 0.46 |
| *PI15* | 8q21.11 | 0.62 | *SNAI1* | 20q13.2 | 0.46 |
| *ADAMTS4* | 1q21-q23 | 0.61 | *P4HA3* | 11q13.4 | 0.46 |
| *FPR2* | 19q13.3-q13.4 | 0.6 | *BCL2A1* | 15q24.3 | 0.45 |
| *CD300E* | 17q25.1 | 0.59 | *CP* | 3q23-q25 | 0.45 |
| *FCAR* | 19q13.42 | 0.57 | *COL5A2* | 2q14-q32 | 0.45 |
| *HAS1* | 19q13.4 | 0.57 | *CXCL6* | 4q13.3 | 0.45 |
| *PXDN* | 2p25 | 0.57 | *HCAR3* | 12q24.31 | 0.45 |
| *GFPT2* | 5q34-q35 | 0.57 | *PDPN* | 1p36.21 | 0.45 |
| *G0S2* | 1q32.2 | 0.57 | *CYP26B1* | 2p13.2 | 0.45 |
| *TREM1* | 6p21.1 | 0.57 | *CLEC4D* | 12p13.31 | 0.45 |
| *CXCL8* | 4q13-q21 | 0.56 | *COL4A1* | 13q34 | 0.44 |
| *IL1B* | 2q14 | 0.55 | *COL3A1* | 2q31 | 0.44 |
| *PAPPA* | 9q33.2 | 0.55 | *CSF2* | 5q31.1 | 0.44 |
| *SOD2* | 6q25.3 | 0.55 | *SLC11A1* | 2q35 | 0.44 |
| *SELE* | 1q24.2 | 0.55 | *CD93* | 20p11.21 | 0.44 |
| *FPR1* | 19q13.4 | 0.54 | *CD200* | 3q13.2 | 0.43 |
| *CXCL1* | 4q21 | 0.54 | *GLT1D1* | 12q24.33 | 0.43 |
| *MMP19* | 12q14 | 0.54 | *THBS1* | 15q15 | 0.43 |
| *TFPI2* | 7q22 | 0.54 | *ADGRG3* | 16q21 | 0.43 |
| *CCL3* | 17q12 | 0.53 | *LOC100507156* | null | 0.43 |
| *ADAM12* | 10q26 | 0.53 | *COL12A1* | 6q12-q13 | 0.42 |
| *ADAMTS3* | 4q13.3 | 0.53 | *GAS1* | 9q21.3-q22 | 0.42 |
| *AQP9* | 15q | 0.51 | *HAS2* | 8q24.12 | 0.42 |
| *HIF1A* | 14q23.2 | 0.51 | *CYR61* | 1p22.3 | 0.42 |
| *OSM* | 22q12.2 | 0.51 | *PDE4B* | 1p31 | 0.42 |
| *IL24* | 1q32 | 0.5 | *CMTM2* | 16q21 | 0.42 |
| *ADAMTS9* | 3p14.1 | 0.5 | *NLRP3* | 1q44 | 0.42 |
| *LIPN* | 10q23.31 | 0.5 | *ADAMTS5* | 21q21.3 | 0.42 |
| *SLC2A3* | 12p13.3 | 0.49 | *DCLK3* | 3p22.2 | 0.42 |
| *TNFAIP3* | 6q23 | 0.49 | *MIR3945* | null | 0.42 |
| *SOCS3* | 17q25.3 | 0.49 | *MIR3606* | null | 0.42 |
| *DIRC1* | 2q33 | 0.49 | *COL6A1* | 21q22.3 | 0.41 |
| *MEDAG* | 13q12.3 | 0.49 | *LIF* | 22q12.2 | 0.41 |
| *CXCR1* | 2q35 | 0.48 | *NNMT* | 11q23.1 | 0.41 |
| *SNAI2* | 8q11 | 0.48 | *PLAU* | 10q22.2 | 0.41 |
| *LILRA5* | 19q13.4 | 0.48 | *PTX3* | 3q25 | 0.41 |
| *COL5A3* | 19p13.2 | 0.48 | *LILRB3* | 19q13.4 | 0.41 |
| *NID2* | 14q22.1 | 0.48 | *C10ORF55* | 10q22.2 | 0.41 |
| *COL15A1* | 9q21-q22 | 0.47 | *RASD1* | 17p11.2 | 0.41 |
| *WISP1* | 8q24.22 | 0.47 | *RASGRP4* | 19q13.1 | 0.41 |
| *ADGRE3* | 19p13.1 | 0.47 | *TMEM45A* | 3q12.2 | 0.41 |
| *GPR84* | 12q13.13 | 0.47 | *CTSL* | 9q21.33 | 0.4 |
| *CXCL2* | 4q21 | 0.4 | *DYSF* | 2p13.3 | 0.4 |
| *IL1R1* | 2q12 | 0.4 | *DOC2B* | 17p13.3 | 0.4 |
| *MEFV* | 16p13.3 | 0.4 | *CCDC71L* | 7q22.3 | 0.4 |
| *RGS16* | 1q25-q31 | 0.4 | *PRR16* | 5q23.1 | 0.4 |
| *PTGS2* | 1q25.2-q25.3 | 0.4 | *NLRP12* | 19q13.42 | 0.4 |
| *SPARC* | 5q31.3-q32 | 0.4 | *UCN2* | 3p21.3 | 0.4 |
| *SIGLEC5* | 19q13.3 | 0.4 |  |  |  |
