# Supplementary material for: Targeting interleukin-6 as a strategy to overcome stroma-induced resistance to chemotherapy in gastric cancer
Source: Mol Cancer. 2019 Mar 30;18:68. doi: 10.1186/s12943-019-0972-8 (PMC6441211; doi:10.1186/s12943-019-0972-8)
Supplement: Supplementary file 6 — Table S2. The functional annotations of co-expressed genes with IL6 in the TCGA gastric cancer dataset. (DOCX 19 kb) [file 12943_2019_972_MOESM6_ESM.docx]

**Table S2** The functional annotations of co-expressed genes with *IL6* in the TCGA gastric cancer dataset

| KEGG pathway | Count | % | *P* value | Gene symbol |
| --- | --- | --- | --- | --- |
| Cytokine-cytokine receptor interaction | 26 | 9.489051095 | 1.52E-09 | *CSF3, CXCL1, CSF2, CCL3, IL1R1, CCL2, PDGFB, CXCL3, CXCL2, TGFB3, CCL8, CXCR1, CXCL6, IL24, PF4V1, CCL4, CCL7, CCL18, IL11, OSM, LIF, INHBA, CCL22, CSF3R, IL1B, IL3RA* |
| Chemokine signaling pathway | 18 | 6.569343066 | 1.72E-06 | *CXCL1, CCL3, CCL2, FGR, CXCL3, CXCL2, NFKBIA, CXCR1, CCL8, GNG11, CXCL6, PF4V1, CCL4, CCL7, STAT3, CCL18, CCL22, SHC3* |
| ECM-receptor interaction | 15 | 5.474452555 | 7.61E-09 | *COL4A2, COL4A1, COL3A1, COL5A3, COL5A2, COL5A1, LAMA4, COL6A3, COL1A2, COL6A2, COL6A1, COL1A1, THBS1, THBS3, FN1* |
| Focal adhesion | 17 | 6.204379562 | 1.98E-05 | *COL4A2, COL4A1, PDGFB, COL3A1, COL5A3, COL5A2, COL5A1, LAMA4, COL6A3, COL1A2, COL6A2, COL6A1, COL1A1, THBS1, SHC3, THBS3, FN1* |
| Pathways in cancer | 17 | 6.204379562 | 0.004823648 | *WNT5A, COL4A2, FGF7, COL4A1, EPAS1, PDGFB, PTGS2, TGFB3, NFKBIA, MMP2, GLI3, STAT3, LAMA4, HIF1A, CSF3R, FGF1, FN1* |
| Jak-STAT signaling pathway | 12 | 4.379562044 | 0.001123492 | *OSM, LIF, CSF3, CSF2, SPRY1, SOCS3, CSF3R, IL24, IL3RA, STAT3, IL11, IL13RA2* |
| Neuroactive ligand-receptor interaction | 11 | 4.01459854 | 0.086047951 | *MCHR1, APLNR, PTGIR, C5AR1, AVPR1A, LPAR4, FPR1, BDKRB1, FPR2, LPAR1, HTR2A* |
| NOD-like receptor signaling pathway | 10 | 3.649635036 | 1.36E-05 | *CXCL1, CCL2, MEFV, CXCL2, CCL8, NFKBIA, IL1B, TNFAIP3, NLRP3, CCL7* |
| Complement and coagulation cascades | 9 | 3.284671533 | 2.14E-04 | *C5AR1, MASP1, C3, SERPINE1, BDKRB1, SERPINA1, C1S, PLAU, PLAUR* |
| Hematopoietic cell lineage | 8 | 2.919708029 | 0.004312 | *CSF3, CSF2, IL1R1, IL1B, CSF3R, IL3RA, CD14, IL11* |
| Toll-like receptor signaling pathway | 7 | 2.554744526 | 0.033620106 | *CCL3, TLR2, NFKBIA, IL1B, LBP, CCL4, CD14* |
| Small cell lung cancer | 6 | 2.189781022 | 0.050345016 | *LAMA4, COL4A2, COL4A1, PTGS2, NFKBIA, FN1* |
